# Indispensable Role of CX_3_CR1^+^ Dendritic Cells in Regulation of Virus-Induced Neuroinflammation Through Rapid Development of Antiviral Immunity in Peripheral Lymphoid Tissues

**DOI:** 10.3389/fimmu.2019.01467

**Published:** 2019-06-27

**Authors:** Jin Young Choi, Jin Hyoung Kim, Ferdaus Mohd Altaf Hossain, Erdenebelig Uyangaa, Seong Ok Park, Bumseok Kim, Koanhoi Kim, Seong Kug Eo

**Affiliations:** ^1^Bio-Safety Research Institute, College of Veterinary Medicine, Chonbuk National University, Iksan, South Korea; ^2^Faculty of Veterinary, Animal and Biomedical Sciences, Sylhet Agricultural University, Sylhet, Bangladesh; ^3^Department of Pharmacology, School of Medicine, Pusan National University, Yangsan-si, South Korea

**Keywords:** CX_3_CR chemokine receptor, Japanese encephalitis, dendritic cells, neuroinflammation, antiviral immunity

## Abstract

A coordinated host immune response mediated via chemokine network plays a crucial role in boosting defense mechanisms against pathogenic infections. The speed of Ag presentation and delivery by CD11c^+^ dendritic cells (DCs) to cognate T cells in lymphoid tissues may decide the pathological severity of the infection. Here, we investigated the role of CX_3_CR1 in the neuroinflammation induced by infection with Japanese encephalitis virus (JEV), a neurotrophic virus. Interestingly, CX_3_CR1 deficiency strongly enhanced susceptibility to JEV only after peripheral inoculation via footpad. By contrast, both CX_3_CR1^+/+^ and CX_3_CR1^−/−^ mice showed comparable susceptibility to JEV following inoculation via intranasal and intraperitoneal routes. CX_3_CR1^−/−^ mice exhibited lethal neuroinflammation after JEV inoculation via footpad route, showing high mortality, morbidity, pro-inflammatory cytokine expression, and uncontrolled CNS-infiltration of peripheral leukocytes including Ly-6C^hi^ monocytes and Ly-6G^hi^ granulocytes. Furthermore, the absence of CX_3_CR1^+^CD11c^+^ DCs appeared to enhance susceptibility of CX_3_CR1^−/−^ mice to JE after peripheral JEV inoculation. CX_3_CR1 ablation impaired the migration of CX_3_CR1^+^CD11c^+^ DCs from JEV-inoculated sites to draining lymph nodes (dLNs), resulting in decreased NK cell activation and JEV-specific CD4^+^/CD8^+^ T-cell responses. However, CX_3_CR1-competent mice showed rapid temporal expression of viral Ags in dLNs. Subsequently, JEV was rapidly cleared, with concomitant generation of antiviral NK cell activation and T-cell responses mediated by rapid migration of JEV Ag^+^CX_3_CR1^+^CD11c^+^ DCs. Using biallelic functional CX_3_CR1 expression system, the functional expression of CX_3_CR1 on CD11c^hi^ DCs appeared to be essentially required for inducing rapid and effective responses of NK cell activation and Ag-specific CD4^+^ T cells in dLNs. Strikingly, adoptive transfer of CX_3_CR1^+^CD11c^+^ DCs was found to completely restore the resistance of CX_3_CR1^−/−^ recipients to JEV, as corroborated by the rapid delivery of JEV Ags in dLNs and attenuation of neuroinflammation in the CNS. Collectively, these results indicate that CX_3_CR1^+^CD11c^+^ DCs play an important role in generating rapid and effective responses of antiviral NK cell activation and Ag-specific T cells after peripheral inoculation with the virus, thereby resulting in conferring resistance to viral infection by reducing the peripheral viral burden.

## Introduction

Japanese encephalitis (JE) is a leading cause of viral encephalitis characterized by extensive neuroinflammation in the central nervous system (CNS) and disruption of the blood-brain barrier (BBB) following infection with JE virus (JEV). The zoonotic, mosquito-borne JEV is a single-stranded, positive-sense RNA virus and is endemic to the Asia-Pacific region, including China, India, and northern Australia (1). Competent vectors for JEV have been recently identified in Germany (2). Notably, porcine transmission of JEV in the absence of mosquitos increases the risk of viral spread and persistence in regions with moderate climate (3). Thus, JEV is becoming a worldwide public health concern. In humans, the clinical presentation of JEV infection ranges from mild febrile illness to severe meningoencephalitis, with nearly 70,000 fatal cases reported annually (4). While most JEV infection in the endemic regions manifests as a mild febrile and subclinical disease that leads to protective immunity, approximately 25–30% of JE cases, involving mostly infants, are lethal and 50% of cases result in permanent neuropsychiatric sequelae (1). Thus, JEV is considered more lethal than the West Nile virus (WNV) infection, which is associated with a fatality rate of 3–5% (1,100 deaths/29,000 symptomatic infections) (5). Vaccination programs are available in endemic regions at risk (6).

Considerable progress in understanding the kinetics and mechanisms of JEV dissemination and JE pathogenesis has been made using murine models (7–9). Following peripheral inoculation of the virus via mosquito bites, JEV initially replicates in peripheral dendritic cells (DCs) and macrophages, eventually invading the CNS through the blood-brain barrier (BBB) (10, 11). JE is considered a neurological and immunopathological disease characterized by uncontrolled hyperimmune response triggered by viral invasion of the CNS (12, 13). While JEV-specific T cells and virus-neutralizing IgM and IgG clear the virus from both peripheral lymphoid tissues and the CNS (14), innate immune response appears to play a critical role in the early control of JEV infection due to delayed adaptive immunity (8, 9, 15). Therefore, type I IFN (IFN-I, typically IFN-α/β) innate immune response is essential for control of JEV. Recent data also indicated that type II IFN (IFN-II, IFN-γ being the only member) produced from NK and CD4^+^ Th1 cells has a positive effect on disease outcome after JEV infection (8, 9).

Coordination between host's innate and adaptive immune response is crucial in regulating infectious diseases caused by various pathogens, including JEV. In particular, the speed at which the host innate and adaptive immune cells respond to infection is critical to the clinical outcome (16). CD11c^+^ dendritic cells (DCs) are professional antigen-presenting cells (APCs) and key instigators of protective immunity (17). Detailed information on the molecular mechanisms underlying the role of CD11c^+^ DCs to initiate protective immunity has solidified their roles in determining the outcomes of infectious diseases (17). The speed that CD11c^+^ DCs deliver and present Ags to cognate T cells in lymph nodes (LNs) through their migration from inflammatory sites may decide the severity of infectious disease. Therefore, understanding the sensitivity of CD11c^+^ DCs in detecting peripheral pathogen invasion and relay of Ags to adaptive immune cells in draining LNs (dLNs) is needed to develop strategies for effective induction of protective immunity.

The speed of host innate immune response including CD11c^+^ DCs to peripheral pathogen infection is controlled by inflammatory mediators such as chemokines (18–20). Chemokine-driven migration of host innate and adaptive immune cells at the periphery and within lymphoid tissues is a key step in the generation of effective protective immunity. Among the members of the chemokine super-family, CX_3_CL1 (fractalkine) belonging to CX_3_C subfamily is unique in that the first two conserved cysteine residues in the chemokine are separated by three non-conserved amino acids (21). CX_3_CL1 is known to exist in two distinct forms: a membrane-anchored form and a soluble form. The soluble CX_3_CL1 acts as a chemoattractant whereas the membrane-anchored CX_3_CL1 functions as an adhesion molecule. CX_3_CL1 is expressed in endothelial cells (22), epithelial cells (22, 23), DCs (24, 25), and neurons (26) upon stimulation by pro-inflammatory cytokines such as IL-1 and TNF-α (27, 28). CX_3_CR1, a CX_3_CL1 receptor, potentially mediates both leukocyte migration and firm adhesion with two distinct expression patterns of CX_3_CL1 (29). CX_3_CR1 is expressed on leukocytes, including monocytes, T-cell subsets, NK cells (30, 31), microglia (32), neurons (33), astrocytes (34), and platelets (35). CX_3_CR1 is also expressed on most tissue macrophages and DCs. It is unlikely to be involved in their ontogeny, homeostatic migration, or colonization of tissues with resident macrophages (36, 37) except kidney DCs (38) and intestinal macrophages (39, 40). CX_3_CR1/CX_3_CL1 axis is involved in the pathophysiology of inflammatory conditions such as cardiovascular disease (41, 42), glomerulonephritis (38), and rheumatoid arthritis (43). Neutralization of CX_3_CL1 improves cardiac function after myocardial infarction (41) and inhibition of CX_3_CR1 reduces atherosclerosis (44). Conversely, studies investigating the protective role of CX_3_CR1 showed an increased risk of liver fibrosis with the loss of CX_3_CR1 in a model of hepatic fibrosis (45). CX_3_CR1 is also required to develop resistance to pulmonary infection by vaccinia virus (46). These findings highlight the complexity of the CX_3_CR1/CX_3_CL1 axis in inflammatory diseases.

However, the role of CX_3_CR1 in the pathogenesis of virus-induced neuroinflammation such as JE has yet to be reported. Therefore, the objective of the present study was to elucidate the role of CX_3_CR1 in neuroinflammation induced by JEV, a neurotrophic virus. In the current study, CX_3_CR1-ablated mice showed increased susceptibility to JE only after peripheral inoculation of JEV infection via footpad, but not intranasally or intraperitoneally. CX_3_CR1 played an important role in the rapid delivery of JEV Ags in dLNs from the peripheral site of infection at an early stage after peripheral JEV inoculation. It also played an important role in viral clearance at the peripheral lymphoid tissues and the CNS by generating effective NK cell and JEV-specific T-cell responses. Furthermore, the delayed migration of CX_3_CR1^+^CD11c^+^ DCs from peripheral site to dLNs appeared to impair NK cell activation and JEV-specific T-cell response in CX_3_CR1-ablated mice. Ultimately, the adoptive transfer of CX_3_CR1^+^CD11c^+^ DCs to CX_3_CR1-ablated mice fully restored the protection against peripheral inoculation of JEV infection. Our results indicate that CX_3_CR1^+^CD11c^+^ DCs are essential to host protection against JE after viral inoculation at the peripheral sites.

## Materials and Methods

### Ethics Statement

All animal experiments described in the present study were conducted at Chonbuk National University according to the guidelines set by the Institutional Animal Care and Use Committee (IACUC) of Chonbuk National University, and were pre-approved by the Ethics Committee for Animal Experiments of Chonbuk National University (approval number: 2013-0028). The animal research protocol used in this study followed the guidelines set up by the nationally recognized Korea Association for Laboratory Animal Sciences (KALAS). All experimental protocols requiring biosafety were approved by the Institutional Biosafety Committee (IBC) of Chonbuk National University.

### Animals, Cells, and Viruses

Wild-type C57BL/6 (H-2^b^) control mice (5–6 weeks old, both female and male) were purchased from SAMTAKO (Osan, Korea), and CX_3_CR1-deficient (CX_3_CR1^−/−^, H-2^b^) mice were obtained from Taconic Biosciences (Rensselaer, NY, USA). The CX_3_CR1^gfp/gfp^ (H-2^b^) mice originally obtained from Jackson laboratory (Bar Harbor, ME, USA) were generously provided by Dr. Doo Hyun Jung (Seoul National University, Seoul, Korea) and crossed with C57BL/6 mice to generate CX_3_CR1^+/gfp^ heterozygous mice. The JEV Beijing-1 strain was propagated in a mosquito cell line C6/36 using DMEM supplemented with 2% FBS, penicillin (100 U/ml), and streptomycin (100 U/ml) as described previously (47). The recombinant vaccinia virus expressing chicken ovalbumin (OVA) was obtained from Dr. Jonathan W. Yewdell (National Institutes of Health, Bethesda, MD, USA) and propagated in CV-1 (American Type Culture Collection, Manassas, VA, USA, CCL70) cell line (48). Virus stocks were titrated using conventional plaque or focus-forming assays and stored in aliquots at −80°C until use.

### Mouse Model of JE

CX_3_CR1^+/+^ and CX_3_CR1^−/−^ mice were infected with JEV [5.0 × 10^7^ plague-forming units (PFU)] via footpad [100 μl, (50 μl/each footpad)], intranasal (20 μl), and intraperitoneal routes (200 μl). Infected mice were monitored daily for mortality, morbidity (weight loss), and neurological disorders (paralysis of front and/or rear limbs, not moving but responsive). Mice were also scored daily for encephalitis signs and symptoms as described previously (49). The encephalitis score represented a progressive range of behaviors: (1) hunched, ruffled fur, (2) altered gait, slow movement, (3) immobile but responsive, (4) moribund and no response, and (5) death.

### Antibodies and Reagents

The following mAbs were obtained from eBioscience (San Diego, CA, USA) or BioLegend (San Diego, CA, USA) for FACS analysis and other experiments: FITC-labeled anti-CD4 (RMA4-5), CD45 (30-F11), CD11b (M1/70), CD3 (145-2C11), and CX_3_CR1 (SA011F11); PE-labeled anti-granzyme B (16G6), CD40L (MR1), CD8 (53–6.7), F4/80 (BM8), IFN-γ (XMG1.2), and CD11c (M1/70); PerCP/Cy5.5-labeled anti-mouse Ly-6C antibody (HK1.4) and IFN-γ (XMG1.2); PE-Cy7-lableled anti-NK1.1 (PK136); APC-labeled anti-Ly6-G (1A8), TNF-α (MP6-XT22), and CD49b-integrin alpha 2 (DX5); and biotin-labeled anti-IL-6 (MP5-32C11) and TNF-α (MP6-XT22). PE-labeled anti-mouse Tmem119 (106-6) was obtained from Abcam (Cambridge, MA, USA). The mAbs against non-structural protein 1 (NS1) and envelope glycoprotein protein (E) of JEV were also obtained from Abcam. The JEV epitope peptide of CD4^+^ T cells [NS3_563−574_ (WCFDGPRTNAIL)] or CD8^+^ T cells [NS4B_215−223_ (9SAVWNSTTA)] was chemically synthesized at Peptron (Daejeon, Korea). Phorbol-12-Myristate-13-Acetate (PMA) and ionomycin were purchased from Sigma-Aldrich (St. Louis, MO, USA).

### Quantitative Real-Time RT-PCR for Determination of Viral Burden and Cytokine Expression

Viral burden and cytokine/chemokine expression in inflammatory and lymphoid tissues were determined via SYBR Green-based real-time qRT-PCR. Mice were infected with JEV (5.0 × 10^7^ PFU) via footpad inoculation and various tissues including popliteal LNs, spleen, and brain were harvested at different time points post-infection (pi). Total RNAs were extracted from the collected tissues using easy-BLUE (iNtRON, Inc., Daejeon, Korea) and subjected to real-time qRT-PCR using a CFX96 Real-Time PCR Detection system (Bio-Rad Laboratories, Hercules, CA, USA). Following reverse transcription of total RNA with High-Capacity cDNA Reverse Transcription Kits (Applied Biosystems, Foster, CA, USA), the reaction mixture (20 μl total) contained 2 μl of template cDNA, 10 μl of 2× SYBR Premix Ex Taq, and 200 nM primers (Supplementary Table 1). These reactions were denatured at 95°C for 30 s and then subjected to 45 cycles of 95°C for 5 s and 60°C for 20 s. After completion of the reaction cycle, the temperature was increased from 65°C to 95°C at the rate of 0.2°C/15 s, and fluorescence was measured every 5 s to construct a melting curve. A control sample lacking template DNA was run with each assay. All measurements were performed at least in duplicate to ensure reproducibility. The authenticity of the amplified product was determined by melting curve analysis. All data were analyzed using Bio-Rad CFX Manager, version 2.1 analysis software (Bio-Rad Laboratories). The expression of cytokines and chemokines was normalized to the levels of housekeeping gene β-actin. Viral burden was expressed by the copy number of viral RNA per microgram of total RNA after calculating the absolute copy number of viral RNA in comparison with the standard cDNA template of viral RNA.

### Histopathological Examinations, Immunohistochemistry, and Confocal Microscopy

Histopathological examination was performed using brains derived from CX_3_CR1^+/+^ and CX_3_CR1^−/−^ mice infected with JEV. Brains were embedded in paraffin at 5 dpi, and 10-μm sections were prepared and stained with H&E. Following deparaffinization, brain sections were also used for the detection of CD11b^+^ myeloid cells by staining with ant-CD11b mAb. After antigen retrieval, endogenous peroxidases were quenched by incubating the slides in 3% H_2_O_2_ for 15 min. The sections were then washed with PBS for 10 min. Endogenous avidin and biotin was blocked using a SuperBlock^TM^ blocking buffer according to manufacturer instructions (Thermo Fisher Sci). The sections were then washed with PBS for 4 min. Primary antibodies (1:100 biotinylated anti-mouse CD11b, eBiosciense) were applied for overnight at 4°C in a humidified chamber. After rinsing the slides in PBS, they were incubated in secondary antibody (1:500 HRP-conjugated streptavidin, eBioscience) for 30 min at room temperature. After washing with PBS for 5 min, color development was achieved by applying diaminobenzidine tetrahydrochloride (DAB) solution (Vector Laboratories) for 0.5–1 min. After washing in distilled water, the sections were counterstained with VECTOR methyl Green (Vector Laboratories), and cover-slipped using a mounting medium (Fisher Scientific). Sections were analyzed using a Nikon Eclipse E600 microscope (Nikon, Tokyo, Japan). For confocal microscopy staining, popliteal LNs and brain were collected at 2 and 5 dpi, respectively, and frozen in optimum cutting temperature (OCT) compound. Sections of 6–7 μm in thickness were cut, air-dried, and fixed with 1:1 mixture of acetone and methanol for 15 min at −20°C. After washing with PBS three times, non-specific binding was blocked with 10% normal goat serum and cells were permeabilized with 0.1% Triton X-100. Staining was performed by incubating sections overnight in moist chambers at 4°C with FITC-conjugated anti-mouse CX_3_CR1, APC-conjugated anti-mouse CD11c, and anti-JEV NS1 and E. Primary antibodies were detected with secondary PE-conjugated goat anti-mouse IgG (SouthernBiotech, Birmingham, AL, USA). Nuclei were counterstained with DAPI (4′6-diamidino-2-phenylindole; Sigma-Aldrich). Fluorescence was observed using a confocal laser scanning microscope (Carl Zeiss, Zena, Germany).

### Cytokine ELISA

Sandwich ELISA was used to determine the levels of IL-6 and TNF-α cytokines in sera. ELISA plates were coated with IL-6 (MP5-20F3) and TNF-α (1F3F3D4) antibodies (eBioscience), and incubated at 4°C overnight. After plates were washed three times with PBS containing 0.05% Tween 20 (PBST), they were blocked with 3% non-fat-dried milk at 37°C for 2 h. Sera and standards for recombinant cytokine proteins (Peprotech, Rehovot, Israel) were added to these plates and incubated at 37°C for 2 h. Plates were washed again with PBST, and then biotinylated IL-6 (MP5-32C11) and TNF-α (polyclonal antibody) antibodies were added. The mixture was incubated overnight at 4°C followed by washing with PBST and subsequent incubation with peroxidase-conjugated streptavidin (eBioscience) at 37°C for 1 h. Color was then developed by adding a substrate (ABTS) solution. Cytokine concentrations were determined using an automated ELISA reader and SoftMax Pro4.3 by comparison with two concentrations of standard cytokine proteins.

### Analysis of Infiltrated Leukocytes in the CNS and Peripheral Lymph Nodes

Mice infected with JEV were perfused with 30 ml of HBSS at 2, 3, and 4 dpi via cardiac puncture of the left ventricle. Brains were then harvested and homogenized by gently pressing them through a 100-mesh tissue sieve, followed by digestion with 25 mg/ml of collagenase type IV (Worthington Biochem, Freehold, NJ, USA), 10 mg/ml DNase I (Amresco, Solon, OH, USA), and incubation with RPMI medium for 1 h at 37°C with shaking. Cells were separated by centrifugation at 800×*g* for 30 min (Axis-Shield, Oslo, Norway) using Opti-prep density gradient (18/10/5%), and the cells were collected from 18 to 10% interface and washed twice with PBS. Leukocytes derived from popliteal LNs and spleen were prepared by gently pressing lymphoid tissues through 100-mesh tissue dishes. The cells were then counted and stained for CD45, CD11b, CD11c, Ly-6C, CX_3_CR1, and Ly-6G with directly conjugated antibodies for 30 min at 4°C. Finally, cells were fixed with 1% formaldehyde. Data collection and analysis were performed using a FACS Calibur flow cytometer (Becton Dickson Medical Systems, Sharon, MA, USA) with FlowJo software (Tree Star, San Carlos, CA, USA).

### Analysis and Activation of NK Cells

The activity of NK cells was assessed by their capacity to produce IFN-γ and granzyme B (GrB) following brief stimulation with PMA and ionomycin (Sigma-Aldrich). Cells were obtained from popliteal LNs of CX_3_CR1^+/+^ and CX_3_CR1^−/−^ mice at 2 dpi and stimulated with PMA and ionomycin in the presence of monensin (2 μM) to induce the expression of IFN-γ (PMA 50 ng/ml plus ionomycin 750 ng/ml for 2 h) or granzyme B (PMA 50 ng/ml plus ionomycin 750 ng/ml for 4 h). The stimulated cells were washed twice with PBS containing monensin and surface-stained with CD3, NK1.1, and DX5 antibodies for 30 min at 4°C. After fixation, cells were washed twice with 1× Permeabilization Buffer (eBioscience) and subjected to intracellular IFN-γ and GrB staining in the buffer for 30 min at room temperature. Stained cells were washed twice with 1× Permeabilization Buffer (eBioscience) and FACS buffer. Analysis was then performed using a FACSCalibur flow cytometer (Becton Dickson Medical Systems) with FlowJo software (Tree Star).

### JEV-Specific Humoral and T-Cell Responses

Humoral responses against JEV were evaluated by JEV-specific IgM and IgG levels in sera using JEV E glycoprotein antigen (Abcam, Cambridge, UK). JEV-specific CD4^+^ and CD8^+^ T-cell responses were determined by intracellular CD154 (also called CD40L), IFN-γ, and TNF-α staining in response to stimulation with JEV epitope peptides. Surviving mice infected with 5.0× 10^7^ PFU JEV were sacrificed on day 7 pi and leukocytes were prepared from popliteal LNs. These leukocytes were cultured in 96-well-culture plates (5 × 10^5^ cells/well) in the presence of synthetic peptide epitopes (NS1_132−145_ and NS4B_215−225_) for 12 h and 6 h to observe CD4 ^+^ and CD8 ^+^ T cell responses, respectively. Monensin at concentration of 2 μM was added to antigen-stimulated cells 6 h before harvest. Cells were washed twice with FACS buffer containing monensin, surface-stained with FITC-anti-CD4 or CD8 antibodies for 30 min at 4°C, and then washed twice with PBS containing monensin. After fixation, cells were washed twice with 1× Permeabilization Buffer (eBioscience) and stained with PepCP-Cy5.5 anti-IFN-γ or APC-anti-TNF-α in the permeabilization buffer for 30 min at room temperature. Intracellular CD154 was detected by addition of CD154 mAb to culture media during peptide stimulation, as described previously (8, 9). Finally, cells were washed twice with PBS and fixed using the fixation buffer. Sample analysis was performed using a FACS Calibur flow cytometer (Becton Dickson Medical Systems) with FlowJo software (Tree Star).

### Purification and Adoptive Transfer of CX_3_CR1^+^CD11c^+^ DCs

CX_3_CR1^+^CD11c^+^ DCs were purified from spleens of CX_3_CR1^+/gfp^ or CX_3_CR1^gfp/gfp^ mice (50). Splenocytes were initially enriched for CD11c^+^ cells using a MACS LS column (Miltenyi Biotec, Bergisch Gladbach, Germany) after surface-staining with PE-conjugated anti-CD11c mAb according to the manufacturer's instructions. Enriched CD11c^+^ cells were then applied to a FACS sorter to purify CX_3_CR1^+^CD11c^+^ DCs. Purified CX_3_CR1^+^CD11c^+^ DCs contained CD11b^+^ cells as well. CX_3_CR1^+/gfp^ and CX_3_CR1^gfp/gfp^CD11c^+^ DCs (5 × 10^5^ cells/mouse, 50 μl) were injected into left and right footpads of CX_3_CR1^−/−^ mice, respectively. CX_3_CR1^−/−^ recipients were immediately infected with JEV (5.0 × 10^7^ PFU) via footpad, and leukocytes were obtained from left and right popliteal LNs at 3 dpi. Popliteal LN cells were surface-stained with PE-conjugated anti-mouse CD11c for CX_3_CR1^+/gfp^ and CX_3_CR1^gfp/gfp^ CD11c^+^ DCs. In some challenge experiments, purified CD11c^+^ DCs (1.5 × 10^6^ cells, 250 μl) were injected i.v. into CX_3_CR1^−/−^ mice. Flow cytometric analysis was performed using a FACS Calibur flow cytometer (Becton Dickson Medical Systems) with FlowJo software (Tree Star).

### CFSE Cell Division Assay in Peripheral LNs

Ag-specific CD4^+^ T cell responses in popliteal LNs were assessed by CFSE cell division following footpad injection of CX_3_CR1^+/gfp^ or CX_3_CR1^gfp/gfp^ CD11c^+^ DCs. Briefly, OVA_323−339_-specific CD4^+^ T cells were purified from OT-II mice using a MACS LS column (Miltenyi Biotec), according to the manufacturer's instructions. Purified OT-II CD4^+^ T cells were labeled with 2.5 μM CFSE and adoptively transferred into CX_3_CR1^−/−^ mice (1 × 10^6^ cells/mouse) injected with CX_3_CR^+/gfp^ and CX_3_CR1^gfp/gfp^ CD11c^+^ DCs in the left and the right footpads, respectively. Three days following infection of CX_3_CR1^−/−^ recipients with recombinant vaccinia virus expressing OVA (1 × 10^6^ PFU/mouse) via footpad route, leukocytes were obtained from left and right popliteal LNs of the recipients and subjected to surface-staining for CD4 and Vα2 using PE-conjugated anti-mouse CD4 and PerCP-conjugated anti-mouse Vα2. Flow cytometric analysis was performed on a FACS Calibur flow cytometer (Becton Dickson Medical Systems) with FlowJo software (Tree Star).

### Statistical Analysis

All data were expressed as average ± standard error of the mean (SEM). Statistically significant differences between groups were analyzed using an unpaired two-tailed Student's *t*-test for *ex vivo* experiments and immune cell analysis. For multiple comparisons, statistical significance was determined using one-way or two-way analysis of variance (ANOVA) with repeated measures followed by Bonferroni *post-hoc* tests. Statistical significance of viral burden and *in vivo* cytokine gene expression were evaluated by Mann-Whitney test or unpaired two-tailed Student's *t*-test. Kaplan-Meier survival curves were analyzed by log-rank test. A *p* ≤ 0.05 was considered significant. All data were analyzed using GraphPadPrism4 software (GraphPad Software, Inc., San Diego, CA, USA).

## Results

### Essential Role of CX_3_CR1 in Conferring Resistance to JEV Following Local, but Not Systemic Infection

**Figure 1:**
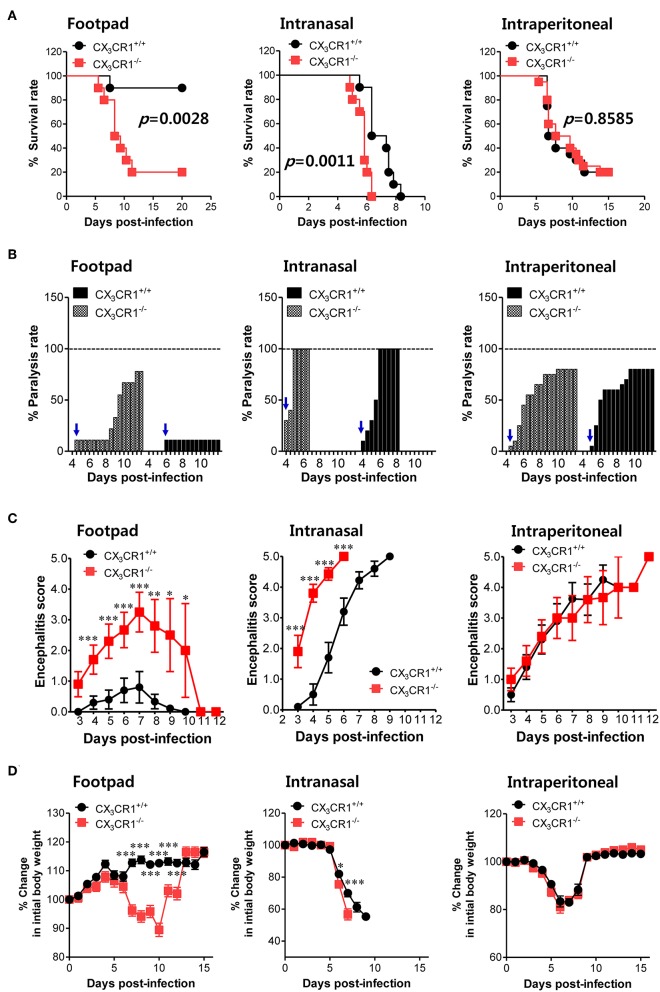
CX_3_CR1 is indispensable for the regulation of JE following local inoculation of virus. **(A)** Susceptibility of CX_3_CR1-ablated mice to JE. Wild-type (CX_3_CR1^+/+^) and CX_3_CR1-deficient (CX_3_CR1^−/−^) mice (5–6 weeks old, *n* = 10–13) were inoculated with JEV (5.0 × 10^7^ PFU) via footpad, intranasal, and intraperitoneal routes. The proportion of surviving mice in each group was monitored daily for 15 or 20 days. **(B)** Ratio of mice showing neurological disorder during JE progression. Mice infected with JEV were examined every 6 h from 4 to 15 dpi and the ratio of mice showing neurological disorder in inoculated mice was recorded. Blue arrows denote a time point of neurological disorder manifestation following JEV infection. **(C)** Encephalitis score. Mice infected with JEV were scored for encephalitis from 3 to 12 dpi. Encephalitis scores were expressed as average score ± SEM of each group. **(D)** Changes in body weight. Changes in body weight were expressed as the average percentage ± SEM of body weight relative to the time of challenge. ^*^p < 0.05; ^**^p < 0.01; and ^***^p < 0.001 for levels between CX_3_CR1^+/+^ and CX_3_CR1^−/−^ mice at indicated dpi.

**Figure 2:**
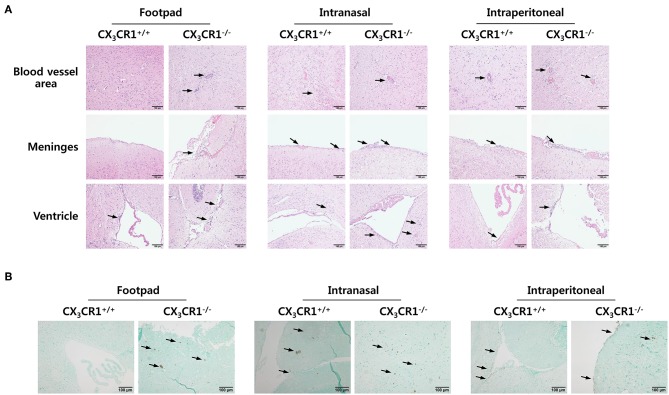
Histopathological analysis supports severe neuroinflammation in the CNS of CX_3_CR1^−/−^ mice. **(A)** Brain sections of CX_3_CR1^+/+^ and CX_3_CR1^−/−^ mice were prepared and stained with H&E 5 days after JEV infection via footpad, intranasal, and intraperitoneal inoculation. Representative photomicrographs of the brain were obtained from blood vessel areas, meninges, and ventricles. **(B)** Detection of infiltrated CD11b^+^ myeloid cells in the brain. Brain sections prepared from CX_3_CR1^+/+^ and CX_3_CR1^−/−^ mice were used for the detection of CD11b^+^ myeloid cells by staining with anti-CD11b mAb. CD11b^+^ cells were detected in cortical parenchyma area of the brain. Images are representative of sections (200×) derived from at least four mice. Interest areas infiltrated with leukocytes are denoted by black arrows.

To investigate the relevance of CX_3_CR1 in JE progression, we infected CX_3_CR1-competent and -deficient mice (CX_3_CR1^+/+^ and CX_3_CR1^−/−^, respectively) with JEV via footpad, intranasal, and intraperitoneal routes. We then compared the susceptibilities of both strains to JE progression. Our data revealed that the ablation of CX_3_CR1 resulted in markedly enhanced susceptibility to JE, with mortality of around 80% after JEV infection via footpad inoculation compared to 10% mortality in CX_3_CR1^+/+^ mice (Figure 1A, *left graph*). In contrast, both CX_3_CR1-competent and deficient mice all succumbed to JE progression following intranasal inoculation of JEV infection, although the survival of CX_3_CR1^+/+^ mice was moderately prolonged (Figure 1A, *middle graph*). A mortality of 80% was observed among CX_3_CR1^+/+^ and CX_3_CR1^−/−^ mice exposed to intraperitoneal inoculation of JEV infection (Figure 1A, *right graph*). These results indicate that CX_3_CR1-ablated mice were highly susceptible to JE progression only after JEV inoculation via footpad route. In support of this finding, the CX_3_CR1^−/−^ mice showed a rapid and higher proportion of neurological disorders starting at 4–5 dpi, compared to CX_3_CR1^+/+^ mice that displayed the delayed signs of neurological disorder around 6–7 days after footpad inoculation of JEV (Figure 1B, *left graph*). However, the proportions of CX_3_CR1^−/−^ mice showing neurological disorder were similar to those of CX_3_CR1^+/+^ mice following intranasal and intraperitoneal inoculation with JEV (Figure 1B, *middle and right graphs*). Furthermore, CX_3_CR1^−/−^ mice scored higher for clinical signs of encephalitis than CX_3_CR1^+/+^ mice after peripheral JEV inoculation (Figure 1C, *left graph*). In contrast, CX_3_CR1^+/+^ and CX_3_CR1^−/−^ mice showed similar kinetics for encephalitis score after intranasal and intraperitoneal inoculation, although CX_3_CR1^−/−^ mice showed rapid clinical signs of encephalitis compared with CX_3_CR1^+/+^ mice (Figure 1C, *middle and right graphs*). CX_3_CR1 ablation resulted in marked changes in body weight during JE progression following footpad inoculation of JEV. However, CX_3_CR1^−/−^ and CX_3_CR1^+/+^ mice showed similar reductions in body weight after intranasal and intraperitoneal JEV administration, except that CX_3_CR1^−/−^ mice showed a slightly higher reduction in body weight at a later stage after intranasal exposure to JEV compared with CX_3_CR1^+/+^ mice (Figure 1D). Similarly, CX_3_CR1^gfp/gfp^ mice with green fluorescent protein (GFP) inserted into two allele of CX_3_CR1 locus showed highly increased susceptibility to JE progression only after peripheral inoculation of JEV via footpad, compared to CX_3_CR1^+/gfp^ mice (Data not shown). To better understand the severity of JE progression in CX_3_CR1^−/−^ mice following peripheral JEV inoculation, we performed histopathological analysis of brains derived from CX_3_CR1^+/+^ and CX_3_CR1^−/−^ mice after JEV inoculation via footpad, intranasal, and intraperitoneal routes. As expected, CX_3_CR1^+/+^ mice showed reduced inflammation involving blood vessels, meninges, and ventricles in the brain compared with CX_3_CR1^−/−^ mice exposed to JEV inoculation via footpad, based on CNS infiltration of peripheral leukocytes (Figure 2A). However, CX_3_CR1^+/+^ and CX_3_CR1^−/−^ mice showed comparable levels of neuroinflammation after intranasal and intraperitoneal inoculation of JEV. CX_3_CR1^+/+^ and CX_3_CR1^−/−^ mice displayed higher peripheral leukocyte infiltration of inflammatory areas after JEV inoculation via intranasal and intraperitoneal routes, compared with CX_3_CR1^+/+^ mice infected via footpad. Enhanced infiltration of CD11b^+^ myeloid cells in the brain of CX_3_CR1^−/−^ mice was further confirmed by immunohistochemistry using anti-CD11b mAb, after JEV inoculation via footpad (Figure 2B). In contrast, CX_3_CR1^+/+^ and CX_3_CR1^−/−^ mice showed no apparent differences in infiltration of CD11b^+^ cells after JEV inoculation via intranasal and intraperitoneal routes. Taken together, our results clearly suggest that CX_3_CR1 ablation leads to severely exacerbated JE progression following peripheral inoculation of JEV, although CX_3_CR1 is dispensable for the control of JE progression upon systemic viral inoculation.

### CX_3_CR1 Regulates Neuroinflammation Following Local JEV Infection

**Figure 3:**
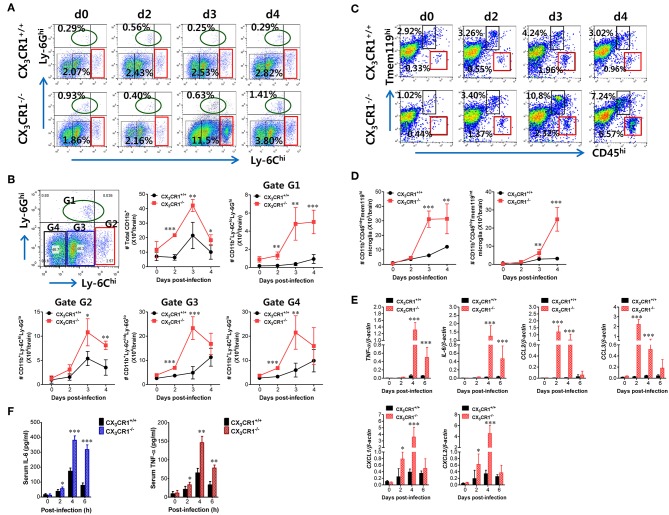
CX_3_CR1 ablation induces higher infiltration of inflammatory leukocytes in the CNS. **(A)** Early and enhanced infiltration of Ly-6C^hi^ monocytes and Ly-6G^hi^ granulocytes in the CNS of CX_3_CR1^−/−^ mice. **(B)** Infiltration kinetics of CD11b^+^ myeloid cell subsets. Values in the dot-plots represent the average percentage of each population after gating on CD45^+^ and subsequent CD11b^+^ cells. **(C,D)** The frequency and number of microglia in the CNS. Infiltrated leukocytes were prepared from the brains of CX_3_CR1^+/+^ and CX_3_CR1^−/−^ mice via vigorous cardiac perfusion and collagenase digestion at indicated dpi. The frequency and absolute number of CD11b^+^CD45^int^Tmem119^hi^ resting microglia and CD11b^+^CD45^hi^Tmem119^int^ activated microglia were determined by flow cytometric analysis. Values in the dot-plots show the average percentage of each population after gating on CD11b^+^ cells. **(E)** Expression of inflammatory cytokines and chemokines in the CNS. Expression of cytokines and chemokines was determined by real-time qRT-PCR using total RNAs extracted from brain tissues at indicated dpi. **(F)** Serum levels of IL-6 and TNF-α. Levels of IL-6 and TNF-α in sera were determined by cytokine ELISA at the indicated dpi. Data show the average ± SEM of levels derived from at least three independent experiments (*n* = 4–5). ^*^p < 0.05; ^**^p < 0.01; and ^***^p < 0.001 for CX_3_CR1^+/+^ vs. CX_3_CR1^−/−^ mice at indicated dpi.

JE is a lethal neuroinflammation characterized by extensive CNS infiltration of myeloid-derived cells including Ly-6C^hi^ monocytes and Ly-6G^hi^ granulocytes (51). Notably, Ly-6C^hi^ monocytes migrate into the infected brain followed by differentiation into DCs, macrophages, and microglia (52, 53). Although the potential contribution of Ly-6C^hi^ monocytes to neuroinflammation remains controversial, CNS infiltration of Ly-6C^hi^ monocytes and Ly-6G^hi^ granulocytes may contribute to the pathophysiology of lethal neuroinflammation (54). To further characterize the exacerbation of JE in CX_3_CR1-ablated mice following peripheral JEV inoculation via footpad, we analyzed CNS infiltration of myeloid-derived cell subsets including monocytes and granulocytes during JE progression. The CX_3_CR1^−/−^ mice showed increased infiltration of Ly-6C^hi^ monocytes and Ly-6G^hi^ granulocytes into the brain compared to CX_3_CR1^+/+^ mice (Figure 3A). The CNS infiltration of Ly-6C^hi^ monocytes in CX_3_CR1^−/−^ mice peaked at 3 dpi and declined subsequently whereas the frequency of Ly-6G^hi^ granulocytes in the CNS of CX_3_CR1^−/−^ mice increased eventually depending on JE progression. CD11b^+^ myeloid cells infiltrating into the brain comprised four subpopulations (G1: Ly-6C^lo^Ly-6G^hi^, G2: Ly-6C^hi^Ly-6G^lo^, G3: Ly-6C^int^Ly-6G^lo^, and G4: Ly-6C^lo^Ly-6G^lo^) depending on the expression of Ly-6C and Ly-6G (Figure 3B). To delineate JE progression in CX_3_CR1^−/−^ mice, we enumerated subpopulations of CNS-infiltrated CD11b^+^ myeloid cells including Ly-6C^hi^Ly-6G^lo^ monocytes and Ly-6C^lo^Ly-6G^hi^ granulocytes. CX_3_CR1-ablated mice harbored a significantly higher number of total CD11b^+^ myeloid cells in the brain during the examination period (0–4 dpi) compared with CX_3_CR1-competent mice (Figure 3B). Similarly, CX_3_CR1 ablation strongly increased CNS infiltration of all the CD11b^+^ subpopulations including Ly-6C^hi^ monocytes and Ly-6G^hi^ granulocytes with saturated levels at 3 dpi. Notably, Ly-6G^hi^ granulocytes were gradually accumulated in the brains of CX_3_CR1^−/−^ mice with a markedly higher level, compared to the CX_3_CR1^+/+^ mice until 4 dpi. It has been reported that microglia contribute to the pathogenesis of encephalitis caused by neurotrophic viruses such as West Nile virus (55) and CNS-infiltrated Ly-6C^hi^ monocytes are differentiated into inflammatory macrophages such as microglia (55). Ly-6C^int^Ly-6G^lo^ and Ly-6C^lo^Ly-6G^lo^ subpopulations in CD11b^+^ myeloid cells may comprise activated microglia cells (56). Therefore, we further examined the changes of resting and activated microglia in the brain based on the expression of Tmem119, a microglia-specific marker (57). As shown in Figure 3C, CX_3_CR1 ablation strongly increased the frequency of CD11b^+^CD45^hi^Tmem119^int^ activated microglia (6.57%) 4 dpi compared with those in CX_3_CR1^+/+^ mice (0.96%). In addition, CX_3_CR1^−/−^ mice contained accumulated number of CD11b^+^CD45^hi^Tmem119^int^ activated microglia in the CNS with markedly higher levels up to 4 dpi, compared to CX_3_CR1^+/+^ mice (Figure 3D). CD11b^+^CD45^int^Tmem119^hi^ resting microglia were also detected in CNS tissues of CX_3_CR1^−/−^ mice with increased levels at 3 dpi compared to CX_3_CR1^+/+^ mice (10.8 vs. 4.24%). This indicates that CX_3_CR1 ablation increased both activated and resting microglia during JE progression.

In terms of severe neuroinflammation involving CX_3_CR1^−/−^ mice following peripheral JEV inoculation via footpad, the expression of cytokines and chemokines within the CNS may further explain encephalitis because neuroinflammation triggered by neurotrophic viruses is indirectly attributed to CNS degeneration by robust immunological responses such as uncontrolled secretion of cytokines and chemokines and the activation of microglia and astrocytes (10, 11, 58). Therefore, we examined the expression of cytokines and chemokines in the CNS. Our results revealed that peripheral JEV inoculation of CX_3_CR1^−/−^ mice strongly increased the expression of TNF-α and IL-6 with peak levels detected at 4 dpi compared with CX_3_CR1^+/+^ mice (Figure 3E). CC chemokines CCL2 and CCL3 were expressed at higher levels (peaks at 2 dpi) in the CNS of CX_3_CR1^−/−^ mice compared with those of CX_3_CR1^+/+^ mice whereas CXC chemokines CXCL1 and CXCL2 showed prolonged and higher expression in the CNS of CX_3_CR1^−/−^ mice until 4 dpi. These results indicate that sequential and uncontrolled expression of cytokines and CC/CXC chemokines might result in severe neuroinflammation in CX_3_CR1^−/−^ mice via infiltration of Ly-6C^hi^ monocytes and Ly-6G^hi^ granulocytes and microglial activation. To further characterize the severity of neuroinflammation in CX_3_CR1^−/−^ mice, we measured the levels of systemic IL-6 and TNF-α. A trend toward rapid induction and increased levels of serum IL-6 and TNF-α in CX_3_CR1^−/−^ mice compared with CX_3_CR1^+/+^ mice was observed (Figure 3F). Taken together, these results demonstrate that robust inflammatory cytokine and chemokine responses drive the severity of neuroinflammation in CX_3_CR1^−/−^ mice.

### Delayed Viral Clearance in Peripheral Lymphoid Tissues Is Closely Associated With JE Exacerbation in CX_3_CR1^−/−^ Mice

**Figure 4:**
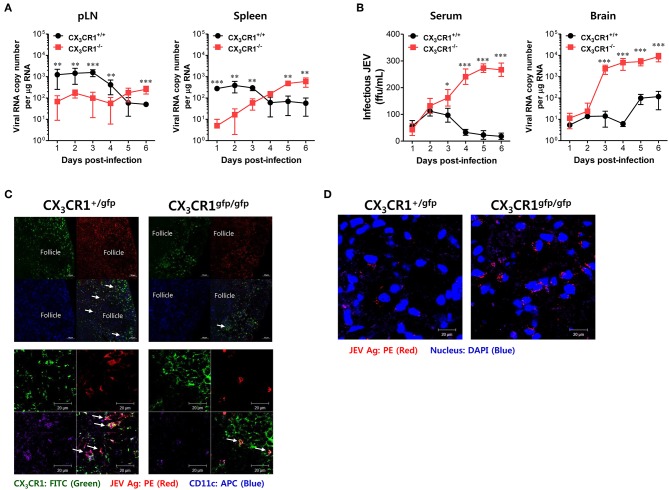
CX_3_CR1 ablation results in delayed viral clearance from peripheral lymphoid tissues. **(A)** Viral burden in peripheral lymphoid during JE progression. **(B)** Infectious JEV in sera and viral burden in CNS tissues during JE progression. Viral burden in popliteal LNs, spleen, and brain of CX_3_CR1^+/+^ and CX_3_CR1^−/−^ mice was assessed by real-time qRT-PCR at the indicated days after infection with JEV via footpad inoculation. Viral RNA load was expressed by viral RNA copy number per microgram of total RNA. The levels of infectious JEV in sera were determined by focus-forming assay. **(C)** Confocal imaging for detection of JEV Ag^+^CX_3_CR1^+^CD11c^+^ DCs in popliteal LNs. Sections of popliteal LNs derived from CX_3_CR1^+/gfp^ and CX_3_CR1^gfp/gfp^ mice infected with JEV via footpad inoculation were co-stained for JEV Ags [E and NS1 (PE red)] and DC marker CD11c (APC purple) at 2 dpi. The CX_3_CR1^gfp^CD11c^+^ DCs co-localizing with JEV Ags in lower magnification images (upper pictures) and higher magnification images (lower pictures) are denoted by white arrows. **(D)** Visualization of JEV in the CNS. Brain sections obtained from JEV-infected CX_3_CR1^+/gfp^ and CX_3_CR1^gfp/gfp^ mice were co-stained with JEV Ags [E and NS1 (PE red)] and nuclear stain DAPI (blue) at 5 dpi. Images are representative of sections derived from at least five mice per group. Data show the average ± SEM of levels derived from at least three independent experiments (*n* = 4–5). ^**^p < 0.01 and ^***^p < 0.001 for CX_3_CR1^+/+^ vs. CX_3_CR1^−/−^ mice at the indicated dpi.

Neurotrophic viruses such as WNV and JEV are thought to replicate in keratinocytes and cutaneous DCs and Langerhans cells following inoculation at peripheral sites such as footpad (59–61). Infected DCs migrate to regional dLNs and seed the virus within these dLNs (59–61). Replication within dLNs leads to primary viremia and subsequent dissemination of infection to permissive organs (such as the spleen) and non-permissive organs (such as kidney and liver) (62). In general, viral replication peaks in the spleen and the serum by 3–4 dpi. Subsequently, viruses are cleared by host defense responses between 6 and 8 dpi (61). However, delayed clearance of infectious virus at peripheral sites by inappropriate host defense may generate large viral loads for CNS invasion. Therefore, we examined the viral burden in dLNs (popliteal LNs), susceptible organs (spleen), sera, and the CNS kinetically during JE progression, in order to elucidate the process of severe neuroinflammation in CX_3_CR1^−/−^ mice following peripheral JEV inoculation via footpad. Somewhat surprisingly, wild-type CX_3_CR1^+/+^ mice carried a higher viral burden in the popliteal LNs and spleen at an early stage until 3–4 dpi compared to CX_3_CR1-ablated mice. Then, these viruses were rapidly cleared at peripheral sites (Figure 4A). However, CX_3_CR1-ablated mice showed a 10- to 100-fold lower viral burden in popliteal LNs and spleen at early stage compared to wild-type mice. Subsequently, viral burden gradually increased depending on JE progression. Ultimately, the CX_3_CR1-ablated mice carried higher viral burdens in popliteal LNs and spleen at late stages compared to CX_3_CR1^+/+^ mice. Due to high viral burden in peripheral lymphoid tissues of CX_3_CR1^−/−^ mice, CX_3_CR1^−/−^ mice showed higher levels of infectious JEV in sera and viral burden in the brain than CX_3_CR1^+/+^ mice (Figure 4B). Notably, CX_3_CR1^−/−^ mice were observed to contain viral burden in brain with around 1,000-fold increased level during JE progression, compared to CX_3_CR1^+/+^ mice. In addition, CX_3_CR1-ablated mice showed a sharp increase in viral burden in the CNS at around 3 dpi, the time point that high viral burden in peripheral lymphoid tissues of CX_3_CR1^+/+^ mice was rapidly decreased. It is thought that JEV inoculated via footpad may be translocated along with infected DCs into dLNs (popliteal LNs) (61). Therefore, we performed confocal microscopy to detect JEV Ags along with DCs in popliteal LNs at the early stage. Viral Ags were detected in the popliteal LNs of CX_3_CR1^+/gfp^ mice with an apparently higher frequency in popliteal LNs at the early stage (2 dpi) compared to CX_3_CR1^gfp/gfp^ mice (Figure 4C, *upper and lower pictures*). Viral Ags were mostly detected within interfollicular and sinus adjacent area near germinal center (Figure 4C, *upper pictures*). Notably, many JEV Ags were co-localized with CX_3_CR1^+^ DCs in interfollicular and T-cell zone in popliteal LNs of CX_3_CR1^+/gfp^ mice. In contrast, JEV Ags were detected in the brains of CX_3_CR1^gfp/gfp^ mice with a high frequency at the late stage (5 dpi) compared to the brains of CX_3_CR1^+/gfp^ mice (Figure 4D). Collectively, these results suggest that CX_3_CR1 plays an important role in the rapid influx of JEV in dLNs (popliteal LNs) at early stage following viral inoculation via footpad. It also plays an important role in viral clearance in the peripheral lymphoid tissues and the CNS at later stage.

### CX_3_CR1 Is Essential for Antiviral NK Cell Activation and Ag-Specific T-Cell Response in dLNs

**Figure 5:**
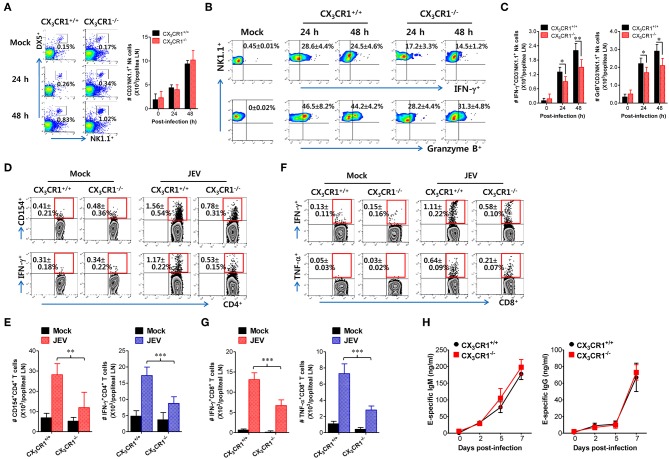
CX_3_CR1 ablation reduced the activation of antiviral NK and JEV-specific CD4^+^/CD8^+^ T cells in peripheral lymphoid tissues. **(A)** NK cell number in popliteal LNs. Leukocytes were obtained from popliteal LNs of CX_3_CR1^+/+^ and CX_3_CR1^−/−^ mice and used to analyze NK cells with flow cytometry at the indicated dpi. Values in dot-plots represent average ± SEM of NK1.1^+^DX5^+^ NK cells after gating on CD3-negative cells (*n* = 4–5). **(B)** NK cell activation. NK cell activation was evaluated by enumerating IFN-γ or granzyme B-producing NK cells with intracellular staining after brief stimulation of leukocytes obtained from popliteal LNs with PMA and ionomycin at 24 and 48 h pi. Values in dot-plots represent the average ± SEM of IFN-γ or granzyme B-producing cells in CD3^−^NK1.1^+^DX5^+^ NK cells (*n* = 4–5). **(C)** Absolute number of IFN-γ or granzyme B-producing NK cells in popliteal LNs. The total number of IFN-γ or granzyme B-producing CD3^−^NK1.1^+^DX5^+^ NK cells was determined by flow cytometric analysis using intracellular and surface staining at 24 and 48 h pi. **(D,E)** JEV-specific CD4^+^ T-cell responses. **(F,G)** JEV-specific CD8^+^ T-cell responses. Leukocytes were obtained from popliteal LNs from surviving CX_3_CR1^+/+^ and CX_3_CR1^−/−^ mice 5 dpi and used for stimulation with JEV epitope peptide of CD4^+^ T cells (NS3_563−574_) or CD8^+^ T cells (NS4B_215−223_) for 12 or 8 h, respectively. The frequency and absolute number of JEV-specific CD4^+^ and CD8^+^ T cells were determined by intracellular CD154 and cytokine (IFN-γ and TNF-α) staining combined with surface CD4 and CD8 staining. **(H)** Serum levels of JEV E protein-specific IgM and IgG. Levels of JEV E protein-specific IgM and IgG in sera (*n* = 7–8) were determined by conventional ELISA using sera collected from surviving mice at 7 dpi. Values in representative dot-plots denote average ± SEM percentage of indicated cell population. Bar charts show the average ± SEM of values derived from at least three independent experiments (*n* = 4–5). ^*^p < 0.05; ^**^p < 0.01; and ^***^p < 0.001 compared between CX_3_CR1^+/+^ and CX_3_CR1^−/−^ mice at indicated dpi.

Antiviral immunity including NK cell activation and JEV-specific T-cell responses is believed to play an important role in regulating JE progression via JEV control and clearance from extraneural tissues (8, 9, 15). The CX_3_CR1-ablated mice showed an impaired clearance of footpad-inoculated JEV in dLNs. Therefore, we compared NK-cell and JEV-specific T-cell responses in popliteal LNs of both wild-type CX_3_CR1^+/+^ and CX_3_CR1^−/−^ mice following footpad JEV inoculation. Both CX_3_CR1^+/+^ and CX_3_CR1^−/−^ mice contained comparable numbers of CD3^−^NK1.1^+^DX5^+^ NK cells in popliteal LNs with increased levels at 24 and 48 h after JEV infection compared to mock-infected mice (Figure 5A). However, CX_3_CR1^−/−^ mice exhibited markedly reduced NK cell activation in popliteal LNs based on the production of IFN-γ and granzyme B from CD3^−^NK1.1^+^DX5^+^ NK cells in response to brief stimulation with PMA and ionomycin (Figure 5B). Similarly, CX_3_CR1^−/−^ mice carried significantly reduced numbers of IFN-γ or granzyme B-producing NK cells in popliteal LNs (Figure 5C). These results indicate that CX_3_CR1 plays an important role in activating NK cells in dLNs following footpad challenge with JEV. Furthermore, we examined JEV-specific CD4^+^ and CD8^+^ T-cell responses in popliteal LNs of surviving CX_3_CR1^+/+^ and CX_3_CR1^−/−^ mice at 5 dpi. CX_3_CR1 ablation resulted in significant reduction of JEV-specific CD4^+^ T-cell responses when CD4^+^ T-cell responses were evaluated by intracellular CD154 and IFN-γ staining in response to stimulation with CD4^+^ T-cell epitope peptide (NS3_563−574_) (Figure 5D). Consistent with this finding, total number of CD154^+^ and IFN-γ^+^ CD4^+^ T cells was found to be significantly decreased in popliteal LNs of CX_3_CR1-ablated mice (Figure 5E). CX_3_CR1-ablated mice also showed reduced numbers of CD8^+^ T cells, based on IFN-γ and TNF-α responses after stimulation with CD8^+^ T-cell epitope peptide (NS4B_215−223_) (Figures 5F,G). In order to further understand that CX_3_CR1 ablation results in impaired NK and T-cell responses, we used a selective, high-affinity inhibitor of CX_3_CR1 (AZD8797) (63). CX_3_CR1^+/+^ wild-type mice were intravenously treated with AZD8797 prior to JEV infection. CX_3_CR1 inhibitor were daily injected to CX_3_CR1^+/+^ mice till analysis date. CX_3_CR1 inhibitor-treated mice displayed highly decreased activation of NK cells in popliteal LNs following JEV inoculation via footpad (Supplementary Figure 1A). Similarly, CX_3_CR1^+/+^ mice showed impaired JEV-specific CD4^+^ and CD8^+^ T-cell responses after treatment with AZD8797 (Supplementary Figures 1B,C), which indicates that functional inhibition of CX_3_CR1 results in impaired generation of NK and JEV-specific T-cell responses in peripheral lymphoid tissues following peripheral JEV inoculation. In addition, we monitored the activation of NK cells in blood during JE progression. As expected, CX_3_CR1^−/−^ mice showed decreased activation of blood NK cells compared to CX_3_CR1^+/+^ mice (Supplementary Figure 2A). Also, CX_3_CR1^−/−^ mice appeared to accumulate lower frequency and number of JEV-specific CD4^+^ and CD8^+^ T cells in the brain, compared to CX_3_CR1^+/+^ mice (Supplementary Figure 2B). However, our data revealed that CX_3_CR1 ablation induced no significant changes in serum IgM or IgG specific for JEV antigen (Figure 5H). This finding indicates that CX_3_CR1 plays no significant role in regulating humoral immune responses specific for JEV Ags. Collectively, these results suggest that CX_3_CR1 plays an important role in NK cell activation and generation of JEV-specific CD4^+^ and CD8^+^ T-cell response in dLNs following peripheral JEV inoculation.

### CX_3_CR1-Ablated Mice Show Impaired Accumulation of CX_3_CR1^+^CD11c^*hi*^ DCs and CX_3_CR1^+^CD11b^*hi*^ Myeloid Cells in dLNs

**Figure 6:**
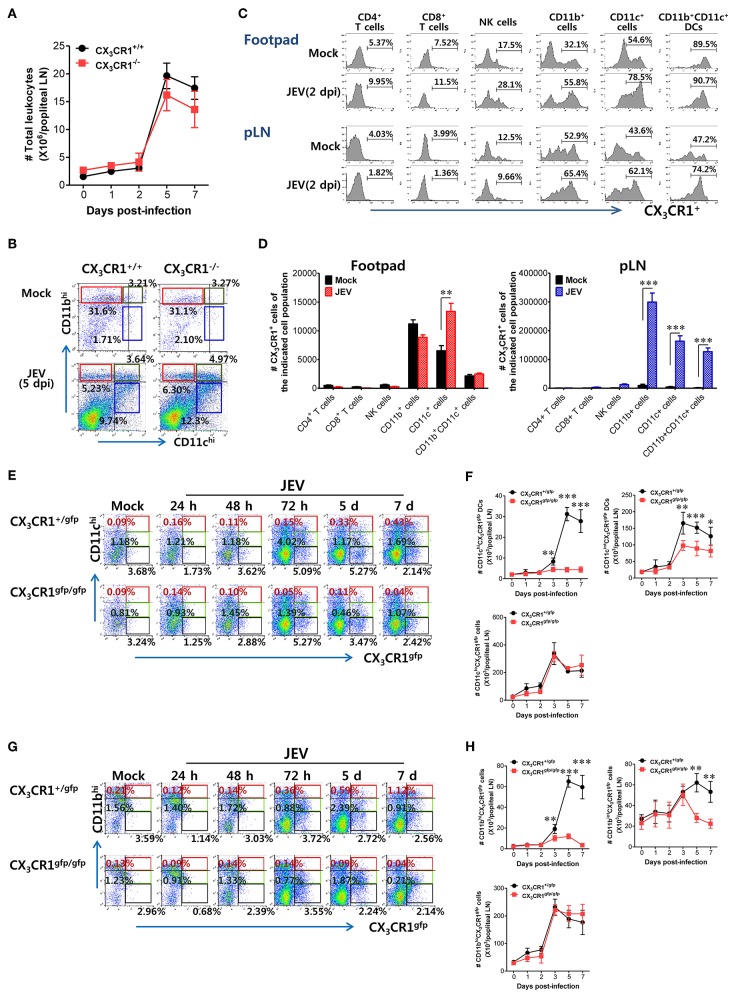
Impaired accumulation of CX_3_CR1^+^ DCs and CD11b^+^ myeloid cells in peripheral lymphoid tissues of CX_3_CR1-ablated mice. **(A)** Total leukocyte number in popliteal LNs. Leukocytes were obtained from popliteal LNs of CX_3_CR1^+/+^ and CX_3_CR1^−/−^ mice, and used in the analysis of total CD45^+^ leukocyte levels at the indicated dpi. **(B)** Analysis of CD11c^+^ and CD11b^+^ myeloid cell population. The proportion of CD11c^+^ and CD11b^+^ myeloid cells was determined by flow cytometric analysis using leukocytes obtained from popliteal LNs of CX_3_CR1^+/+^ and CX_3_CR1^−/−^ mice 5 dpi. **(C)** CX_3_CR1 expression in leukocyte subpopulations. **(D)** Changes of CX_3_CR1^+^ cell number in leukocyte subpopulations. Leukocytes were prepared from footpad and popliteal LNs with collagenase digestion and surface-stained by CX_3_CR1 mAb combined with CD3, CD45, CD11c, CD11b, CD4, CD8, DX5, and NK1.1 mAbs at 0 and 2 dpi. Values in histograms denote the average percentage ± SEM of CX_3_CR1-positive cells in indicated leukocyte subpopulations, including CD3^+^CD4^+^, CD3^+^CD8^+^, CD3^−^NK1.1^+^DX5^+^, CD45^+^CD11b^+^, CD45^+^CD11c^+^, and CD45^+^CD11b^+^CD11c^+^ cells. **(E,F)** Delayed accumulation of CX_3_CR1^gfp^ DCs in popliteal LNs of CX_3_CR1^gfp/gfp^ mice. Leukocytes were obtained from popliteal LNs via collagenase digestion at the indicated dpi and used to determine CX_3_CR1^gfp^ DC subsets (CX_3_CR1^gfp^CD11c^hi^, CX_3_CR1^gfp^CD11c^int^) and CX_3_CR1^gfp^CD11c^lo^ cells through flow cytometric analysis, after infecting CX_3_CR1^+/gfp^ and CX_3_CR1^gfp/gfp^ mice with JEV via footpad inoculation. **(G,H)** Impaired accumulation of CX_3_CR1^gfp^ myeloid cells in popliteal LNs of CX_3_CR1^gfp/gfp^ mice. CX_3_CR1^gfp^ myeloid cells including CX_3_CR1^gfp^CD11b^hi^ and CX_3_CR1^gfp^CD11b^int^ cells in popliteal LNs were counted by flow cytometric analysis from 1 to 7 dpi. Values in representative dot-plots denote the average ± SEM percentage of the indicated cell population after gating on CD45^+^ cells, while bar charts show the average ± SEM of values derived from at least three independent experiments (*n* = 4–5). ^*^p < 0.05; ^**^p < 0.01; and ^***^p < 0.001 for CX_3_CR1^+/+^ vs. CX_3_CR1^−/−^ mice at indicated dpi.

CX_3_CR1 and its ligand CX_3_CL1 contribute to immune cell recruitment during inflammation via either chemotaxis or adhesion because the CX_3_CR1-CX_3_CL1 axis is known to play a role in the migration of NK cells, T cells, monocytes, and mast cells (29). CX_3_CR1 ablation failed to alter the total number of CD45^+^ leukocytes in popliteal LNs following JEV infection via footpad inoculation (Figure 6A). Notably, both CX_3_CR1^+/+^ and CX_3_CR1^−/−^ mice showed rapid and comparable increase in leukocyte levels in dLNs from 2 dpi. Also, CD11c^+^ and CD11b^+^ myeloid cells were detected in popliteal LNs of CX_3_CR1^+/+^ and CX_3_CR1^−/−^ mice with comparable levels 5 dpi (Figure 6B). These data indicate that CX_3_CR1 ablation did not affect the migration of CD45^+^ leukocytes as well as CD11c^+^ and CD11b^+^ myeloid cells. CX_3_CR1 is expressed on several types of leukocytes, including monocytes, T-cell subsets, NK cells, microglia, neurons, astrocytes, and platelets (30–35). To delineate the leukocyte subpopulation whose migration is affected by CX_3_CR1 during JE progression, we examined the expression of CX_3_CR1 in various subsets of immune cells recruited in popliteal LNs and footpad following footpad inoculation of JEV. As a result, we found that CD11b^+^ and CD11c^+^ myeloid cell populations showed constitutive expression of CX_3_CR1 at higher levels compared to other immune cells, including NK cells, CD4^+^, and CD8^+^ T cells (Figure 6C). The CX_3_CR1 expression in CD11b^+^ and CD11c^+^ cells derived from footpad and popliteal LNs was strongly increased following JEV infection. In support, CX_3_CR1^+^ cells in CD11c^+^ and CD11b^+^ myeloid cell populations were more accumulated in popliteal LNs compared to other immune cells (Figure 6D). The CD11b^+^ and CD11c^+^ myeloid cells mainly include antigen-presenting cells such as CD11b^+^CD11c^+^ myeloid DCs (64, 65). CX_3_CR1^−/−^ mice showed impaired NK cell activation and JEV-specific T-cell responses. Indeed, CX_3_CR1-positive cells in CD11b^+^CD11c^+^ DC population were likely to be recruited into popliteal LNs at 2 days following footpad inoculation of JEV (Figure 6C). Therefore, we examined the kinetics of migration of CX_3_CR1^gfp^CD11c^+^ DCs and CD11b^+^ myeloid cells into popliteal LNs of CX_3_CR1^+/gfp^ and CX_3_CR1^gfp/gfp^ mice following footpad inoculation. CX_3_CR1^gfp/gfp^ mice displayed markedly impaired recruitment of CX_3_CR1^gfp^CD11c^hi^ and CX_3_CR1^gfp^CD11c^int^ DCs in popliteal LNs following footpad challenge (Figure 6E). Similarly, CX_3_CR1^gfp/gfp^ mice accumulated a lower number of CX_3_CR1^gfp^CD11c^hi^ and CX_3_CR1^gfp^CD11c^int^ DCs in popliteal LNs during JE progression compared to CX_3_CR1^+/gfp^ mice (Figure 6F). Notably, CX_3_CR1^gfp^CD11c^hi^ DCs were detected with very low levels in popliteal LNs of CX_3_CR1^gfp/gfp^ mice. Consistent with impaired recruitment of CX_3_CR1^gfp^CD11c^hi^ DCs in CX_3_CR1^gfp/gfp^ mice, the recruitment of CX_3_CR1^gfp^CD11b^hi^ and CD11b^int^ myeloid cells into the popliteal LNs of CX_3_CR1^gfp/gfp^ mice was delayed (Figures 6G,H). However, the total numbers of CX_3_CR1^gfp^CD11c^lo^ and CD11b^lo^ cells were comparable between CX_3_CR1^+/gfp^ and CX_3_CR1^gfp/gfp^ mice, suggesting that the recruitment of CX_3_CR1^gfp^CD11c^lo^ and CD11b^lo^ cells was unlikely to depend on the CX_3_CR1-CX_3_CL1 axis. In support, treatment with CX_3_CR1 inhibitor resulted in delayed accumulation of CX_3_CR1^+^CD11c^hi^ and CD11c^int^ DCs in popliteal LNs following JEV inoculation via footpad, while migration of CX_3_CR1^+^CD11c^lo^ cells was not affected by CX_3_CR1 inhibitor (Supplementary Figure 3A). Accumulation of CX_3_CR1^+^CD11b^hi^ and CD11b^int^ myeloid cells was also reduced by treatment of CX_3_CR1 inhibitor (Supplementary Figure 3B). Collectively, these results indicate that functional inhibition of CX_3_CR1 results in the impaired recruitment of CX_3_CR1^+^CD11c^+^ DCs and CX_3_CR1^+^CD11b^+^ myeloid cells into the dLNs following footpad inoculation of JEV. In particular, impaired recruitment of CX_3_CR1^+^CD11c^hi^ DCs into dLNs of CX_3_CR1^−/−^ mice appears to decrease NK cell activation and JEV-specific T-cell response, compared to CX_3_CR1^+/+^ mice.

### CX_3_CR1-Ablated DCs Exhibit Delayed and Reduced NK-Cell Activation and CD4^+^/CD8^+^ T Cell Response in dLNs

**Figure 7:**
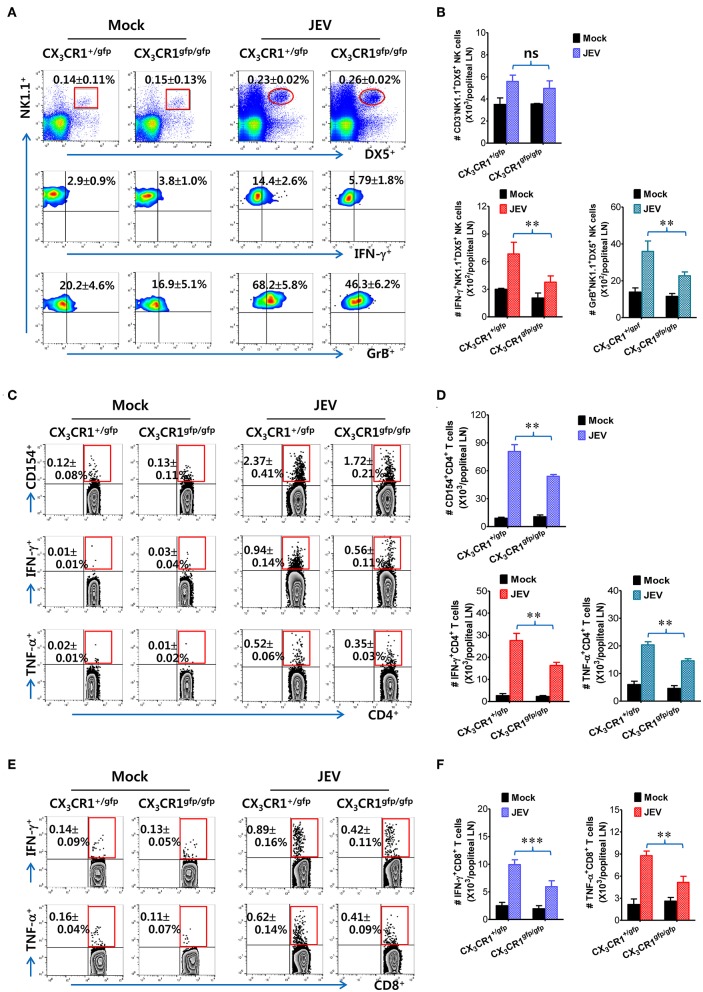
Delayed and reduced NK cell activation and JEV-specific CD4^+^/CD8^+^ T-cell responses by CX_3_CR1-ablated DCs. **(A)** Frequency and activation of NK cells in popliteal LNs of CX_3_CR1^−/−^ recipients of CX_3_CR1-ablated DCs. CX_3_CR1^gfp^CD11c^+^ DCs from spleens of CX_3_CR1^+/gfp^ or CX_3_CR1^gfp/gfp^ mice were sorted and injected into CX_3_CR1^−/−^ recipient mice via footpad (5 × 10^5^ cells/mouse). CX_3_CR1^−/−^ recipients were subsequently infected with JEV via footpad inoculation. Two days following infection, the frequency and activation of NK cells in popliteal LNs were determined by intracellular staining for IFN-γ and granzyme B (GrB) along with surface staining for CD3, NK1.1, and DX5 following brief stimulation with PMA and ionomycin. Values in the plots represent the average ± SEM of IFN-γ or GrB-producing cells in NK1.1^+^ cells after gating on CD3^−^NK1.1^+^DX5^+^ NK cells (*n* = 4–5). Vaginal leukocytes unstimulated with PMA and ionomycin were used for negative control. **(B)** Absolute number of IFN-γ or granzyme B-producing CD3^−^NK1.1^+^DX5^+^ NK cells in popliteal LNs of CX_3_CR1^−/−^ recipients. **(C,D)** JEV-specific CD4^+^ T-cell responses in popliteal LNs of CX_3_CR1^−/−^ recipients. **(E,F)** JEV-specific CD8^+^ T-cell responses in popliteal LNs of CX_3_CR1^−/−^ recipients. At 7 days after JEV infection, leukocytes were obtained from popliteal LNs of CX_3_CR1^−/−^ recipient mice injected with CX_3_CR1^+/gfp^ or CX_3_CR1^gfp/gfp^ DCs and used for stimulation with JEV epitope peptide of CD4^+^ T cells (NS3_563−574_) or CD8^+^ T cells (NS4B_215−223_) for 12 or 8 h, respectively. The frequency and absolute number of JEV-specific CD4^+^ and CD8^+^ T cells were determined by intracellular CD154 and cytokine (IFN-γ and TNF-α) staining combined with surface staining for CD4 and CD8. Values in representative dot-plots represent the average ± SEM percentage of the indicated cell population. Bar charts show the average ± SEM of values derived from at least three independent experiments (*n* = 4–5). ^**^p < 0.01 and ^***^p < 0.001 comparing the indicated groups.

It has been reported that CX_3_CR1^gfp/gfp^ mice with GFP inserted into two alleles of the CX_3_CR1 locus show no functional expression of CX_3_CL1 receptor. However, all surface CX_3_CR1-positive cells in heterozygous CX_3_CR1^+/gfp^ mice show GFP expression as well as biallelic functional expression of CX_3_CL1 receptor (50). It is believed that CD11c^+^ DCs play a crucial role in activating NK cells via NK-DC crosstalk and in initiating Ag-specific CD4^+^ and CD8^+^ T-cell responses (66). However, our results provided no direct evidence that delayed recruitment of CX_3_CR1^+^CD11c^+^ DCs resulted in the impaired NK cell activation and JEV-specific T-cell responses in popliteal LNs of CX_3_CR1^−/−^ mice. To directly demonstrate the role of delayed CX_3_CR1^+^CD11c^+^ DC recruitment in impaired NK cell activation and T-cell responses, the CX_3_CR1^gfp^CD11c^+^ DCs were purified from the spleens of CX_3_CR1^gfp/gfp^ or CX_3_CR1^+/gfp^ mice and subsequently injected into footpads of CX_3_CR1^−/−^ mice. We then examined NK cell activation in popliteal LNs of CX_3_CR1^−/−^ recipient mice at 2 days following immediate JEV infection via footpad inoculation. CX_3_CR1^−/−^ mice injected with CX_3_CR1^+/gfp^ or CX_3_CR1^gfp/gfp^CD11c^+^ DCs showed comparable frequencies of CD3^−^NK1.1^+^DX5^+^ NK cells in popliteal LNs following footpad inoculation of JEV (Figure 7A). However, CX_3_CR1^−/−^ mice injected with CX_3_CR1^gfp/gfp^CD11c^+^ DCs showed markedly reduced activation of NK cells compared to CX_3_CR1^−/−^ mice injected with CX_3_CR1^+/gfp^CD11c^+^ DCs, when NK cell activation was evaluated by IFN-γ and granzyme B production in response to brief stimulation by PMA and ionomycin. CX_3_CR1^−/−^ recipients of CX_3_CR1^+/gfp^ and CX_3_CR1^gfp/gfp^CD11c^+^ DCs also carried comparable numbers of NK cells in popliteal LNs. However, footpad injection of CX_3_CR1^gfp/gfp^CD11c^+^ DCs into CX_3_CR1^−/−^ mice resulted in a significant reduction of IFN-γ and granzyme B-producing NK cells compared to CX_3_CR1^−/−^ recipients of CX_3_CR1^+/gfp^CD11c^+^ DCs (Figure 7B). Furthermore, we examined the JEV-specific CD4^+^ and CD8^+^ T-cell responses in popliteal LNs of CX_3_CR1^−/−^ mice injected with CX_3_CR1^+/gfp^ and CX_3_CR1^gfp/gfp^CD11c^+^ DCs at 7 days after footpad inoculation of JEV. CX_3_CR1^−/−^ recipients of CX_3_CR1^gfp/gfp^CD11c^+^ DCs showed lower responses of CD4^+^ T cells specific for JEV Ag compared to CX_3_CR1^−/−^ recipients of CX_3_CR1^+/gfp^CD11c^+^ DCs, based on frequencies of CD154^+^, IFN-γ^+^, and TNF-α^+^ cells in CD4^+^ T cells stimulated with CD4^+^ T-cell epitope (NS3_563−574_) (Figure 7C). Similarly, CX_3_CR1^−/−^ recipients of CX_3_CR1^gfp/gfp^CD11c^+^ DCs harbored significantly decreased numbers of JEV-specific CD4^+^ T cells in popliteal LNs compared to CX_3_CR1^−/−^ recipients of CX_3_CR1^+/gfp^CD11c^+^ DCs (Figure 7D). Consistent with the weak response of JEV-specific CD4^+^ T cells in popliteal LNs of CX_3_CR1^−/−^ recipients of CX_3_CR1^gfp/gfp^CD11c^+^ DCs, footpad injection of CX_3_CR1^gfp/gfp^CD11c^+^ DCs induced a lower level of JEV-specific CD8^+^ T cell response in popliteal LNs compared to CX_3_CR1^−/−^ mice injected with CX_3_CR1^+/gfp^CD11c^+^ DCs (Figures 7E,F). Ultimately, these results indicate that functional deficiency of CX_3_CR1 expression in CD11c^+^ DCs leads to impaired NK cell activation and reduced the generation of JEV-specific CD4^+^ and CD8^+^ T-cell response in dLNs following footpad injection.

### CX_3_CR1-Ablated DCs Show Delayed Initiation of Ag-Specific CD4^+^ T-Cell Responses in dLNs

**Figure 8:**
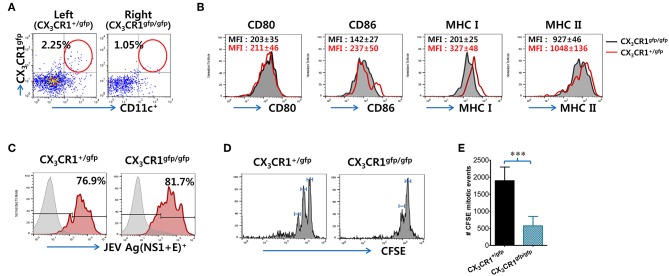
Functional expression of CX_3_CR1 on CD11c^+^ DCs is required for their recruitment from infection sites to draining LNs. **(A)** Recruitment of CX_3_CR1^+/gfp^ and CX_3_CR1^gfp/gfp^ DCs in popliteal LNs of CX_3_CR1^−/−^ recipients. Sorted CX_3_CR1^+/gfp^ and CX_3_CR1^gfp/gfp^ DCs were injected into CX_3_CR1^−/−^ mice via left and right footpads, respectively. Leukocytes were obtained from popliteal LNs of CX_3_CR1^−/−^ recipients via collagenase digestion at 3 dpi and used for flow cytometric analysis to detect CX_3_CR1^+/gfp^ or CX_3_CR1^gfp/gfp^CD11c^+^ DCs. **(B)** Phenotypes of CX_3_CR1^+/gfp^ and CX_3_CR1^gfp/gfp^ DCs recruited in popliteal LNs of CX_3_CR1^−/−^ recipients. The phenotypic levels of CX_3_CR1^+/gfp^ and CX_3_CR1^gfp/gfp^ DCs were determined by flow cytometric analysis using leukocytes obtained from popliteal LNs of CX_3_CR1^−/−^ recipients at 3 dpi. **(C)** JEV Ags expression in CX_3_CR1^+/gfp^ and CX_3_CR1^gfp/gfp^ DCs recruited in popliteal LNs of CX_3_CR1^−/−^ recipients. The expression of JEV Ags E and NS1 in CX_3_CR1^+/gfp^ and CX_3_CR1^gfp/gfp^ DCs recruited in popliteal LNs of CX_3_CR1^−/−^ recipients was determined by intracellular staining for JEV E and NS1 protein combined with surface staining for CX_3_CR1-gfp and CD11c. **(D)** *In vivo* proliferation of Ag-specific CD4^+^ T cells in popliteal LNs of CX_3_CR1^−/−^ recipients. CX_3_CR1^−/−^ recipient mice injected with purified and CFSE-labeled OT-II CD4^+^ T cells were infected with vaccinia virus expressing OVA (1 × 10^6^ PFU) via footpad inoculation after injection with CX_3_CR1^+/gfp^ and CX_3_CR1^gfp/gfp^ DCs into left and right footpads (5 × 10^5^ cells/footpad), respectively. The proliferation of OT-II CD4^+^ T cells in popliteal LNs of each corresponding leg was evaluated by CFSE division 3 dpi. **(E)** Total mitotic events of CFSE-labeled OT-II CD4^+^ T cells. Total mitotic events were determined by subtracting the number of precursors from the number of daughters generated by each precursor population based on CFSE division. Values in representative dot-plots or histograms represent the average ± SEM percentage of the indicated cell population. Bar charts show the average ± SEM of values derived from at least three independent experiments (*n* = 4–5). ^***^p < 0.001 comparing the indicated groups.

Because impaired NK cell activation and JEV-specific T-cell responses were observed in popliteal LNs of CX_3_CR1^−/−^ recipients injected with CX_3_CR1^gfp/gfp^CD11c^+^ DCs, we determined whether functional deficiency of CX_3_CR1 expression affected their migration from injection site (footpad) to popliteal LNs following JEV infection, resulting in impaired NK cell activation and JEV-specific T-cell responses. CX_3_CR1^+/gfp^ and CX_3_CR1^gfp/gfp^CD11c^+^ DCs were sorted from spleens of heterozygous CX_3_CR1^+/gfp^ and homozygous CX_3_CR1^gfp/gfp^ mice, and injected into left and right footpads of CX_3_CR1^−/−^ mice, respectively. Recruitment of CX_3_CR1^+/gfp^ and CX_3_CR1^gfp/gfp^CD11c^+^ DCs in popliteal LNs of CX_3_CR1^−/−^ recipients was then observed at 3 days after footpad inoculation of JEV. Our results revealed that functional deficiency of CX_3_CR1 expression delayed the migration of CD11c^+^ DCs from the footpad to popliteal LNs, as the lower frequency of CX_3_CR1^gfp/gfp^CD11c^+^ DCs was observed in popliteal LNs of the right footpad injected with CX_3_CR1^gfp/gfp^CD11c^+^ DCs compared to popliteal LNs of the left footpad injected with CX_3_CR1^+/gfp^CD11c^+^ DCs (Figure 8A). However, CX_3_CR1^+/gfp^ and CX_3_CR1^gfp/gfp^CD11c^+^ DCs recruited in popliteal LNs of CX_3_CR1^−/−^ recipients showed similar expression of phenotype markers including CD80, CD86, MHC I, and MHC II (Figure 8B). Also, JEV Ags showed similar levels in CX_3_CR1^+/gfp^ and CX_3_CR1^gfp/gfp^CD11c^+^ DCs derived from the popliteal LNs of left and right footpads, respectively (Figure 8C). These data indicate that functional deficiency of CX_3_CR1 expression affected their migration from infection site to dLNs, but not phenotypic changes and JEV infectivity at the peripheral site. Therefore, the delayed delivery of JEV Ags by functional CX_3_CR1 deficiency in CD11c^+^ DCs could induce delayed and weak JEV-specific T-cell responses in dLNs. To quantitatively determine this possibility, we used transgenic OT-II CD4^+^ T cells that recognize OVA_323−339_ epitopes derived from chicken ovalbumin (OVA). The CFSE-labeled OT-II CD4^+^ T cells purified from OT-II mice were adoptively transferred into CX_3_CR1^−/−^ mice, followed by footpad inoculation with vaccinia virus expressing OVA after injection of CX_3_CR1^+/gfp^ and CX_3_CR1^gfp/gfp^CD11c^+^ DCs into the left and right footpads, respectively. It was found that OT-II CD4^+^ T cells in popliteal LNs of the left footpad injected with CX_3_CR1^+/gfp^CD11c^+^ DCs proliferated rapidly compared to OT-II CD4^+^ T cells in popliteal LNs of the right footpad injected with CX_3_CR1^gfp/gfp^CD11c^+^ DCs (Figure 8D). In addition, total mitotic events of OT-II CD4^+^ T cells occurring in popliteal LNs (67) showed a 4-fold increase in the OT-II CD4^+^ T cells of popliteal LNs in the left footpad injected with CX_3_CR1^+/gfp^CD11c^+^ DCs compared to OT-II CD4^+^ T cells of popliteal LNs in the right footpad injected with CX_3_CR1^gfp/gfp^CD11c^+^ DCs (Figure 8E). These results suggest that the rapid Ag-specific T cell response in dLNs is mediated via functional expression of CX_3_CR1 in CD11c^+^ DCs.

### Adoptive Transfer of CX_3_CR1^+^ DCs Ameliorates JE

**Figure 9:**
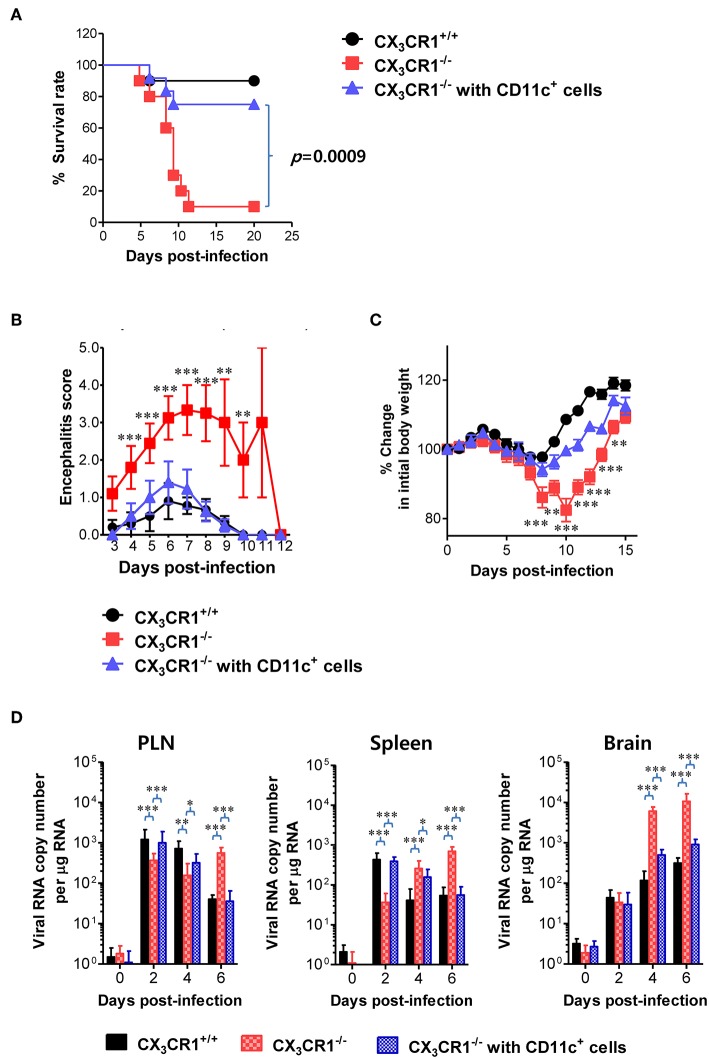
Restoration of resistance to JE by adoptive transfer of CX_3_CR1^+^ DCs. CX_3_CR1^+^CD11c^+^ DCs from spleens of wild-type mice were sorted and adoptively transferred into CX_3_CR1^−/−^ mice via tail vein and foot pad inoculation (5 × 10^5^ cells/mouse). CX_3_CR1^−/−^ recipients (*n* = 10–11) were subsequently infected with JEV (5.0 × 10^7^ PFU) via footpad inoculation. CX_3_CR1^+/+^ wild-type mice and CX_3_CR1^−/−^ mice that received no cells were used as positive and negative controls, respectively. **(A)** Susceptibility of CX_3_CR1^−/−^ recipients for CX_3_CR1^+^CD11c^+^ DCs to JE. The proportion of surviving mice in each group was monitored daily for 20 days. **(B)** Encephalitis score. Mice infected with JEV were scored for encephalitis from 3 to 12 dpi and the encephalitis score was expressed as the average score ± SEM of each group. **(C)** Changes in body weight. Changes in body weight were expressed as average percentage ± SEM of body weight relative to the time of challenge. **(D)** Viral burden in peripheral lymphoid and CNS tissues of CX_3_CR1^−/−^ recipients for CX_3_CR1^+^CD11c^+^ DCs during JE progression. The viral burdens in spleen, brain, and spinal cord of CX_3_CR1^−/−^ recipients infected with JEV were assessed by real-time qRT-PCR at indicated dpi. Viral RNA load was expressed as viral RNA copy number per microgram of total RNA. Data show the average ± SEM of levels derived from at least three independent experiments (*n* = 4–5). ^*^p < 0.05; ^**^p < 0.01; and ^***^p < 0.001 comparing CX_3_CR1^−/−^ mice and CX_3_CR1^−/−^ recipients of CD11c^+^ DC at indicated dpi.

A functional deficiency of CX_3_CR1 expression in CD11c^+^ DCs abrogated the rapid induction of NK cell activation and JEV-specific T-cell responses, thereby providing enhanced susceptibility to JEV peripheral inoculation. Therefore, we investigated whether adoptive transfer of CX_3_CR1^+^CD11c^+^ DCs to CX_3_CR1^−/−^ mice could restore protection against JEV infection inoculated via footpad. CX_3_CR1^+^CD11c^+^ DCs were purified from spleens of wild-type CX_3_CR1^+/+^ mice and adoptively transferred into CX_3_CR1^−/−^ mice before JEV infection via footpad. Strikingly, CX_3_CR1^−/−^ recipients of CX_3_CR1^+^CD11c^+^ DCs showed fully recovered resistance to JE caused by peripheral JEV inoculation. Their resistance levels were comparable to CX_3_CR1^+/+^ mice (Figure 9A). Adoptive transfer of CX_3_CR1^+^CD11c^+^ DCs to CX_3_CR1^−/−^ recipients strongly enhanced the resistance to JE with a mortality of around 20%, compared to CX_3_CR1^−/−^ mice that showed 90% mortality. CX_3_CR1^−/−^ recipients of CX_3_CR1^+^CD11c^+^ DCs also showed clinical scores for encephalitis comparable to CX_3_CR1^+/+^ wild-type mice whereas CX_3_CR1^−/−^ mice not injected with CX_3_CR1^+^CD11c^+^ DCs showed a higher encephalitis score (Figure 9B). CX_3_CR1^−/−^ mice injected with CX_3_CR1^+^CD11c^+^ DCs also showed lower changes in body weight compared to CX_3_CR1^−/−^ mice (Figure 9C). Wild-type CX_3_CR1^+/+^ mice showed rapid dissemination of JEV to popliteal LNs and spleens at the early stage following footpad inoculation of JEV. Subsequently, the virus was rapidly cleared. However, CX_3_CR1^−/−^ mice showed a gradual increase in viral burden in the dLNs and CNS. Therefore, we kinetically examined the viral burden in dLNs, spleen, and CNS of CX_3_CR1^−/−^ mice, depending on JE progression. Interestingly, CX_3_CR1^−/−^ recipients of CX_3_CR1^+/+^CD11c^+^ DCs showed elevated viral burdens in popliteal LNs and spleen with levels comparable to wild-type CX_3_CR1^+/+^ mice at the early stage, and subsequently the rapid clearance of virus occurred in the peripheral lymphoid tissues eventually (Figure 9D). CX_3_CR1^−/−^ mice injected with CX_3_CR1^+/+^CD11c^+^ DCs also showed lower viral burdens in the CNS during JE progression compared to CX_3_CR1^−/−^ mice. In conclusion, these results indicate that reconstitution of CX_3_CR1^−/−^ mice with CX_3_CR1^+^CD11c^+^ DCs restore protection against peripheral JEV infection.

### Attenuation of JE Progression by Adoptive Transfer of CX_3_CR1^+^ DCs

**Figure 10:**
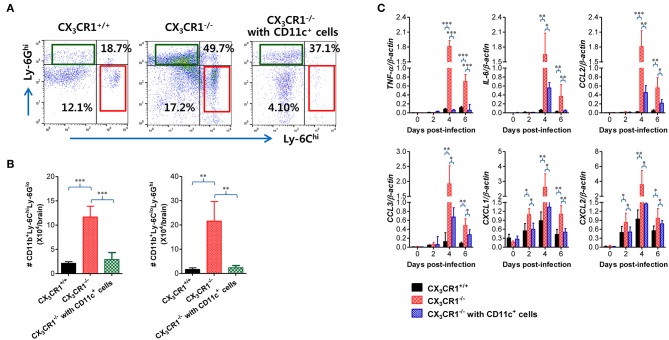
Adoptive transfer of CX_3_CR1^+^ DCs attenuates JE progression. **(A)** Attenuated infiltration of Ly-6C^hi^ monocytes and Ly-6G^hi^ granulocytes in the CNS of CX_3_CR1^−/−^ mice injected with CX_3_CR1^+^CD11c^+^ DCs. **(B)** Accumulated number of infiltrated Ly-6C^hi^ monocytes and Ly-6G^hi^ granulocytes in the CNS of CX_3_CR1^−/−^ mice injected with CX_3_CR1^+^CD11c^+^ DCs. CNS-infiltrated leukocytes were obtained from the brains of CX_3_CR1^−/−^ recipients of CX_3_CR1^+^CD11c^+^ DCs with vigorous cardiac perfusion and collagenase digestion at 3 dpi. CX_3_CR1^+/+^ wild-type mice and CX_3_CR1^−/−^ mice that received no cells were used as positive and negative controls, respectively. Values in the dot-plots represent the average percentage of each population after gating on CD45^+^ and subsequent CD11b^+^ cells. **(C)** Expression of inflammatory cytokines and chemokines in the CNS of CX_3_CR1^−/−^ mice injected with CX_3_CR1^+^CD11c^+^ DCs. Expression of cytokines and chemokines was determined by real-time qRT-PCR using total RNA extracted from brain tissue at indicated dpi. Data show the average ± SEM of levels derived from at least three independent experiments (*n* = 4–5). ^*^p < 0.05; ^**^p < 0.01; and ^***^p < 0.001 comparing CX_3_CR1^−/−^ mice and CX_3_CR1^−/−^ recipients of CX_3_CR1^+^CD11c^+^ DC at indicated dpi.

In order to further characterize the neuroinflammation of CX_3_CR1^−/−^ recipients injected with CX_3_CR1^+^CD11c^+^ DCs, we examined CNS infiltration of Ly-6C^hi^ monocytes and Ly-6G^hi^ granulocytes in CX_3_CR1^−/−^ recipients during JE progression. Our results revealed that CX_3_CR1^−/−^ mice injected with CX_3_CR1^+/+^CD11c^+^ DCs showed a lower CNS infiltration of Ly-6C^hi^ monocytes and Ly-6G^hi^ granulocytes following footpad inoculation of JEV compared with CX_3_CR1^−/−^ mice (Figure 10A). Notably, CNS infiltration of Ly-6C^hi^ monocytes was markedly reduced in CX_3_CR1^−/−^ recipients injected with CX_3_CR1^+/+^CD11c^+^ DCs, compared to CX_3_CR1^−/−^ mice not injected with CX_3_CR1^+/+^CD11c^+^ DCs. Similarly, CX_3_CR1^−/−^ recipients injected with CX_3_CR1^+^CD11c^+^ DCs showed lower Ly-6C^hi^ monocytes and Ly-6G^hi^ granulocytes in the CNS compared to CX_3_CR1^−/−^ mice (Figure 10B). Furthermore, we examined the expression of inflammatory cytokines and chemokines in the CNS of CX_3_CR1^−/−^ recipients injected with CX_3_CR1^+^CD11c^+^ DCs. CX_3_CR1^−/−^ mice reconstituted with CX_3_CR1^+^CD11c^+^ DCs showed a diminished expression of cytokines (TNF-α, IL-6) and chemokines (CCL2, CCL3, CXCL1, and CXCL2) in the CNS during JE progression (Figure 10C). These results indicate that reconstitution of CX_3_CR1^−/−^ mice with CX_3_CR1^+^CD11c^+^ DCs ameliorated JE progression.

## Discussion

Our results demonstrate that CX_3_CR1 is essential for the regulation of neuroinflammation in the CNS following peripheral inoculation of JEV infection via footpad, but not intranasal or intraperitoneal inoculation of JEV. Interesting finding in the present study was that CX_3_CR1^+^CD11c^hi^ DCs were strongly correlated with increased susceptibility of CX_3_CR1-ablated mice to JE after peripheral JEV inoculation. Several lines of evidence support the essential role of CX_3_CR1^+^CD11c^hi^ DCs in providing resistance to JE. First, the rapid appearance of JEV Ag in dLNs of CX_3_CR1^+/+^ mice was closely associated with recruitment of CX_3_CR1^+^CD11c^hi^ DCs, which effectively induced NK cell activation and JEV-specific CD4^+^ T-cell responses. In contrast, impaired recruitment of CX_3_CR1^+^CD11c^hi^ DCs delayed and weakened NK cell activation and JEV-specific CD4^+^ T cells in dLNs of CX_3_CR1^−/−^ mice. Second, using biallelic functional expression system of CX_3_CR1, our results revealed that the functional expression of CX_3_CR1 on CD11c^hi^ DCs was required to induce rapid and effective NK cell activation and CD4^+^ T-cell responses in dLNs. Injection of CX_3_CR1^+/gfp^CD11c^+^ DCs into footpads of CX_3_CR1^−/−^ mice resulted in complete activation of NK cells and CD4^+^ T-cell responses in dLNs whereas injection of CX_3_CR^gfp/gfp^CD11c^+^ DCs resulted in impaired and weak NK cell activation and CD4^+^ T cells. Finally, the adoptive transfer of CX_3_CR1^+^CD11c^+^ DCs was found to fully restore the resistance of CX_3_CR1^−/−^ mice to JE. Adoptive transfer of CX_3_CR1^+^CD11c^+^ DCs attenuated JE progression following peripheral JEV inoculation. Collectively, our results indicate that CX_3_CR1^+^CD11c^+^ DCs play an important role in generating rapid and effective NK cell activation and Ag-specific CD4^+^ T-cell responses after viral inoculation at peripheral sites, thereby inducing resistance to viral diseases.

DCs are key players in the initiation and generation of Ag-specific CD4^+^ and CD8^+^ T-cell responses. They also mediate the activation of NK cells via DC-NK crosstalk (16, 17, 66). CX_3_CR1 is expressed on various leukocyte subsets, including monocytes, DCs, macrophages, microglia, and specific memory T cells (30–35, 68). Indeed, our results revealed that CX_3_CR1 was strongly expressed on CD11b^+^ and CD11c^+^ leukocytes, especially CD11b^+^CD11c^+^ DC population, compared to CD4^+^, CD8^+^ T, and NK cells. CD11b^+^F4/80^+^ macrophages and CD11c^+^ DCs exhibit different migratory properties. CD11c^+^ DCs migrate from peripheral tissues to dLNs to interact with T cells and induce immune responses whereas macrophages largely remain in tissues (69). We analyzed the antiviral immune responses of NK cell activation and JEV-specific CD4^+^/CD8^+^ T cells in dLNs following peripheral inoculation of JEV. CX_3_CR1 ablation reduced NK cell activation and T-cell responses specific for JEV Ag in the dLNs, which was closely associated with delayed viral clearance in lymphoid tissues (LNs and spleen). Although CX_3_CR1^+/+^ mice showed higher viral burdens temporally in lymphoid tissues at the early stage (1–3 dpi), JEV was rapidly cleared in the peripheral tissues. This viral clearance in the peripheral lymphoid tissues of CX_3_CR1^+/+^ mice appeared to be mediated by rapid and effective NK cell activation and JEV-specific CD4^+^ and CD8^+^ T cell response. The rapid delivery of viral Ag to cognate T cells might induce the prompt proliferation of viral Ag-specific CD4^+^ and CD8^+^ T cells in dLNs (17, 66, 69). IFN-γ produced from CD4^+^ and CD8^+^ T cells is considered to play a role in recovery from primary infection with JEV (70). NK cells might involve in regulating JE progression through reducing viral burden via IFN-γ production and their cytolytic action, because early activation of NK cells has been associated with mild clinical diseases following viral infection (71). Also, CX_3_CR1-dependent recruitment of mature NK cells into the CNS may play a certain role in controlling neuroinflammation (72, 73), even though the contribution of NK cells in the CNS was not addressed in the present study. Because JEV is already replicating at a high level by 3 dpi in CX_3_CR1^−/−^ mice, NK cell activation is more plausible in the early control of viral replication at the peripheral sites than T-cell responses, which take time to develop. The early appearance of antiviral CD4^+^ and CD8^+^ T-cell responses in CX_3_CR1^+/+^ mice is likely to effectively prevent virus from invading in the CNS at the later stage (4–7 dpi) during JE progression.

To assess the cell type involved in rapid NK cell activation and JEV-specific T-cell response in dLNs of CX_3_CR1-competent mice, the expression of CX_3_CR1 on various leukocytes in both dLNs and inoculation site was analyzed. The results showed that ~90% of CD11b^+^CD11c^+^ DC populations at the inoculation site (footpad) were CX_3_CR1-positive. The CX_3_CR1^+^CD11b^+^CD11c^+^ DC population was rapidly recruited to dLNs following peripheral JEV inoculation, because the proportion of CX_3_CR1^+^ cells in CD11b^+^CD11c^+^ DC population was increased from 40% to around 70–80%. CX_3_CR1 ablation also interfered with the migration of CD11c^+^ DCs and CD11b^+^ myeloid cells into dLNs (popliteal LNs). The impaired migration of CX_3_CR1^+^CD11c^+^ DCs from the inoculation site to dLNs in CX_3_CR1^−/−^ mice was likely to induce delayed and diminished responses of JEV-specific CD4^+^ and CD8^+^ T cells. The CX_3_CR1^−/−^ mice also showed a weak activation of NK cells without changes in the absolute number of CD3^−^NK1.1^+^DX5^+^ NK cells in dLNs. This finding was corroborated by IFN-γ and granzyme B-producing NK cells. It is plausible that the impaired migration of CD11c^+^ DCs affected the activation of NK cells in dLNs because DCs play a crucial role in activating NK cells via DC-NK crosstalk (66). DCs located in various tissues manifest diverse phenotypes and functional expression depending on the context of tissues. Conventional CD11c^+^ DCs (cDC) originating in common DC precursors (CDPs) via cDC-restricted progenitors (pre-cDCs) have been detected in lymphoid and non-lymphoid tissues. They are strategically located in areas to actively detect signs of pathogens and damage in the cellular and physiological environment (74). Until now, the two main subtypes of developmentally distinct cDCs include cDC1 and cDC2, with distinct tissue-specific expression of CX_3_CR1 (74). For example, the cDC1 and cDC2 subtypes in the spleen express CX_3_CR1 with subtle differences, depending on cDC subtypes and their developmental transcription factors (74). CX_3_CR1^+^CD11b^+^ DCs stimulate the protective effector T-cell response in the intestine whereas CD103^+^CX3CR1^−^ DCs mediate Treg responses to ingested Ags and commensal organisms (75). Similarly, in the present study, migratory CX_3_CR1^+^CD11c^+^ DCs appear to mediate the generation of effector CD4^+^ and CD8^+^ T-cell responses, to prevent CNS dissemination of JEV by reducing the viral burden at the peripheral sites. Furthermore, IL-12 production mediated by CX_3_CR1^+^ cDC1 in the spleen is necessary to induce IFN-γ synthesis by NK cells and CD4^+^ Th1 differentiation (76–78). These studies reinforce our findings suggesting that CX_3_CR1^+^CD11c^+^ DCs enhanced NK cell activation and Ag-specific CD4^+^ Th1 and CD8^+^ T cells in dLNs after injection into footpad. Using biallelic functional expression system of CX_3_CR1 (footpad injection of CX_3_CR^+/gfp^ and CX_3_CR1^gfp/gfp^CD11c^+^ DCs) and transgenic OT-II CD4^+^ T cells, we analyzed the role of CX_3_CR1^+^CD11c^+^ DCs in generating rapid and effective NK cell activation and Ag-specific CD4^+^ Th1 responses. CX_3_CR1-CX_3_CL1 axis not only mediates the migration of leukocytes to promote cell-to-cell interaction with an inflamed endothelium, but also regulates the development of monocytes and their survival (79). We did not investigate whether the survival of CD11c^+^ DCs was dependent on CX_3_CR1 following JEV infection. CD11c^+^ DCs are permissible for replication of JEV RNA but not productive for their progeny virus (80). While the migration of JEV Ag-bearing CX_3_CR1^+^ DCs could drive increased viral titers in dLNs, the differences of CX_3_CR1-competent and incompetent DCs in the capture of viral Ag might be potential reason for inducing reduced JEV-specific T-cell responses in dLNs, thereby resulting in exacerbated outcomes of diseases (81, 82). However, the present study suggest that CX_3_CR1 is unlikely to mediate JEV replication and viral Ag capture in CD11c^+^ DCs because CX_3_CR1^+/gfp^ and CX_3_CR1^gfp/gfp^CD11c^+^ DCs carry comparable levels of JEV Ag (NS1 and E protein) in dLNs. Also, CX_3_CR1^+/gfp^, and CX_3_CR1^gfp/gfp^CD11c^+^ DCs show comparable expression of phenotype markers related to Ag-presentation CD80, CD86, MHC I, and MHC II), indicating that both CX_3_CR1-competent and incompetent DCs have the same ability to present Ags. Therefore, CX_3_CR1 expression on CD11c^+^ DCs appeared to involve in their migration from inoculation site to dLNs, which subsequently generated effective NK cell activation and Ag-specific T-cell responses. CX_3_CR1^+/gfp^CD11c^+^ DCs were detected at a higher frequency in the corresponding dLNs, compared to CX_3_CR1^gfp/gfp^CD11c^+^ DCs. Enhanced migration of CX_3_CR1^+^CD11c^+^ DCs carrying JEV Ags might increase the binding frequency to cognate CD4^+^ and CD8^+^ T cells in dLNs, thereby inducing rapid and robust T-cell responses as well as NK cell activation. The striking evidence supporting the regulatory role of CX_3_CR1^+^CD11c^+^ DCs in JE progression was based on the adoptive transfer of purified CX_3_CR1^+^CD11c^+^ DCs into CX_3_CR1^−/−^ recipients. CX_3_CR1^−/−^ mice injected with CX_3_CR1^+^CD11c^+^ DCs displayed resistance to JE with a survival rate comparable to CX_3_CR1^+/+^ wild-type mice after peripheral JEV inoculation. Our data revealed that CX_3_CR1^−/−^ mice injected with CX_3_CR1^+^CD11c^+^ DCs showed rapid expression of JEV RNA in dLNs and the spleen at the early stage after JEV inoculation. Subsequently, these viruses were rapidly cleared from the peripheral lymphoid tissues as shown in CX_3_CR1^+/+^ wild-type mice. This finding strongly suggests that CX_3_CR1^+^CD11c^+^ DCs provides resistance to JE via rapid and effective NK cell activation and Ag-specific CD4^+^/CD8^+^ T-cell responses with rapid delivery of viral Ag in peripheral lymphoid tissues.

Because all JEV in dLNs appears not to be delivered by trafficking CX_3_CR1^+^ DCs, our data may discount the role of other Ag-capturing cells in delivery of viral Ags from inoculation sites to blood and the spleen, such as sinus lining CD169^+^ macrophages. Sinus lining CD169^+^ macrophages are known to be responsible for the capture of pathogens and are frequently the first cell type infected in the spleen and dLNs (83). Furthermore, because viral Ags were detected in the spleen within 1 dpi, it is assumed that there is abundant lymph-borne virus passing through dLN. Lymph-borne JEV is likely to be captured by subcapsular sinus macrophages that also express CX_3_CR1, thereby providing viral Ags to CD11c^+^ DCs with cross-presentation to activate T cells. Indeed, JEV Ags were mostly detected within interfollicular and sinus adjacent area near germinal center, where DCs and sinus lining macrophages are co-located. The interaction between CD169^+^ macrophages and CD11c^+^ DCs is believed to play an important role in generating effective Ag-specific T-cell responses in dLNs (83). The role of CD169^+^ sinus lining macrophages in delivery of viral Ags and subsequent generation of Ag-specific T-cell responses via cross-presentation was not addressed in this study. Sinus lining macrophages are reported to play a role in limiting the dissemination of neutrophic viruses including WNV at the early stage but are not required for the generation of WNV-specific CD8^+^ T-cell responses in dLNs (84). Therefore, the role of sinus lining macrophages in generating T-cell responses against neurotrophic viruses such as WNV and JEV remains defined.

CX_3_CR1-CX_3_CL1 axis plays an important role in facilitating adhesion and transmigration of Ly-6C^hi^ monocytes as CX_3_CR1 is highly expressed on Ly-6C^hi^ monocytes (79). Recently, a direct and evolutionarily conserved role has been suggested for CX_3_CR1-CX_3_CL1 interactions in monocyte survival (79). Thus, functional ablation of CX_3_CR1 on Ly-6C^hi^ monocytes might affect their migration from peripheral sites into the CNS. A debatable issue in the present study was that the functional ablation of CX_3_CR1 expression on Ly-6C^hi^ monocytes did not affect their migration into the CNS because CX_3_CR1-ablated mice contained increased number of Ly-6C^hi^ monocytes in the brain. Chemokine responses can be redundant, although sequential responses are needed for selective and tailored environment of Ly-6C^hi^ monocytes (85, 86). It is plausible that CCR2-CCL2 axis might compensate for the migration of Ly-6C^hi^ monocytes into the CNS for CX_3_CR1 deficiency, because CCR2 mediates the recruitment of Ly-6C^hi^ monocytes to inflamed tissues (87, 88). In fact, we detected higher levels of CCL2 expression in the CNS of CX_3_CR1^−/−^ mice compared to CX_3_CR1^+/+^ mice. A large load of JEV disseminated from peripheral sites to the CNS may strongly induce the expression of chemokines including CCL2 in the CNS, suggesting that the control of viral replication at peripheral site is important to suppress neuroinflammation caused by peripheral JEV inoculation.

Our findings contrast with the detrimental role of CX_3_CR1 in sterile inflammatory conditions such as atopic dermatitis (89), glomerulonephritis (38), and collagen-induced arthritis (43). In particular, the exclusive CX_3_CR1-dependent migration of kidney DCs promotes glomerulonephritis progression (38). In contrast, CX_3_CR1 is thought to exert a protective role in kidney fibrosis (90), steatohepatitis (91), and parasite-induced hepatic granuloma formation (92). The protective role of CX_3_CR1 in inflammatory diseases has been attributed to regulation of macrophage differentiation, proliferation, and intestinal homeostasis, without focusing on the role of CX_3_CR1 in leukocyte migration (90–92). These complex roles of CX_3_CR1 in regulating inflammatory diseases are likely to depend on disease types (93). Bonduelle et al. demonstrated that CX_3_CR1 played an important role in providing protective immunity against pulmonary infection with vaccinia virus, but NK cell activation and Ag-specific T-cell responses through rapid CX_3_CR1-dependent delivery of viral Ags in dLNs were not addressed in their study (46). Our results strongly support the protective role of CX_3_CR1 through rapid migration of CD11c^+^ DCs to present viral Ag in dLNs. Therefore, the role of CX_3_CR1 in JE progression after intranasal and intraperitoneal inoculation of JEV infection might be discounted due to the lack of dLNs or CNS in proximity to the injection site. In conclusion, because CX_3_CR1 deficiency promotes neuroinflammation induced by neurotrophic viruses such as JEV and WNV infection following peripheral inoculation, CX_3_CR1 inhibition should be carefully considered when treating sterile inflammation in diseases such as multiple sclerosis (63), atopic dermatitis (89), and glomerulonephritis (38).

## Ethics Statement

All animal experiments described in the present study were conducted at Chonbuk National University according to the guidelines set by the Institutional Animal Care and Use Committee (IACUC) of Chonbuk National University, and were pre-approved by the Ethics Committee for Animal Experiments of Chonbuk National University (approval number: 2013-0028). The animal research protocol used in this study followed the guidelines set up by the nationally recognized Korea Association for Laboratory Animal Sciences (KALAS). All experimental protocols requiring biosafety were approved by the Institutional Biosafety Committee (IBC) of Chonbuk National University.

## Author Contributions

JC, JK, and SE conceived and designed this research. JC, JK, FH, EU, and SP performed animal study design, analysis, and interpretation. JC and EU performed Ag-specific T-cell responses. BK and KK provided critical discussion for histopathological investigations and key resources. JC, JK, and SE performed data interpretation and wrote the draft of the manuscript. All authors reviewed the manuscript.

### Conflict of Interest Statement

The authors declare that the research was conducted in the absence of any commercial or financial relationships that could be construed as a potential conflict of interest.

## Abbreviations

APCs: antigen-presenting cells
BBB: blood-brain barrier
CNS: central nervous system
DCs: dendritic cells
dLNs: draining lymph nodes
dpi: days post-infection
IFN-I: type I interferon
IFN-II: type II interferon
JEV: Japanese encephalitis virus
WNV: West Nile virus.
