# Supplementary material for: Indispensable Role of CX3CR1+ Dendritic Cells in Regulation of Virus-Induced Neuroinflammation Through Rapid Development of Antiviral Immunity in Peripheral Lymphoid Tissues
Source: Front Immunol. 2019 Jun 27;10:1467. doi: 10.3389/fimmu.2019.01467 (PMC6610490; doi:10.3389/fimmu.2019.01467)
Supplement: Supplementary file 1 [file Data_Sheet_1.PDF]

Indispensable role of CX_3CR1^+ dendritic cells in regulation of virus-induced neuroinflammation through rapid development of antiviral immunity in peripheral lymphoid tissues

Jin Young Choi[1], Jin Hyoung Kim[1], Ferdaus Mohd Altaf Hossain[1,2], Erdenebelig Uyangaa[1], Seong Ok Park[1], Bumseok Kim[1], Koanhoi Kim[3], Seong Kug Eo[1*]

[1]College of Veterinary Medicine and Bio-Safety Research Institute, Chonbuk National University, Iksan 54596, Republic of Korea
[2]Faculty of Veterinary, Animal and Biomedical Sciences, Sylhet Agricultural University, Sylhet, Bangladesh
[3]Department of Pharmacology, School of Medicine, Pusan National University, Yangsan 50612, Republic of Korea

Supplementary Table 1. Real-time qRT-PCR primers for cytokines, chemokines, and JEV.

Gene name[a]	Primer sequence (5'-3')[b]	Position cDNA	Gene Bank ID
IL-6	FP: TGG GAA ATC GTG GAA ATG AG	209-228	NM_031168
	RP: CTC TGA AGG ACT CTG GCT TTG	442-462	
TNF-α	FP: CGT CGT AGC AAA CCA CCA AG	438-457	NM_013693
	RP: TTG AAG AGA ACC TGG GAG TAG ACA	564-587	
CCL2	FP: AAA AAC CTG GAT CGG AAC CAA	347-367	NM_011333
	RP: CGG GTC AAC TTC ACA TTC AAA G	426-447	
CCL3	FP: CCA AGT CTT CTC AGC GCC AT	158-177	NM_011337.2
	RP: GAA TCT TCC GGC TGT AGG AGA AG	206-228	
CXCL1	FP: CGC TGC TGC TGG CCA CC	101-120	NM_008176.3
	RP: GGC TAT GAC TTG GGT TTG GG	245-264	
CXCL2	FP: ATC CAG AGC TTG AGT GTG ACG C	194-215	NM_009140.2
	RP: AAG GCA AAC TTT TTG ACC GC	264-283	
JEV	FP: GGC TTA GCG CTC ACA TCC A	4132–4150	AB920399.1
	RP: GCT GGC CAC CCT CTC TTC TT	4207–4226	
β-actin	FP: TGG AAT CCC TGT GGG ACC ATG AAA C	885-909	NM_007393.3
	RP: TAA AAC GCA GCT CAG TAA CAG TCC G	1209-1233	

[a] IL, interleukin; TNF-α, tumor necrosis factor-α

[b] FP, forward primer; RP, reverse primer

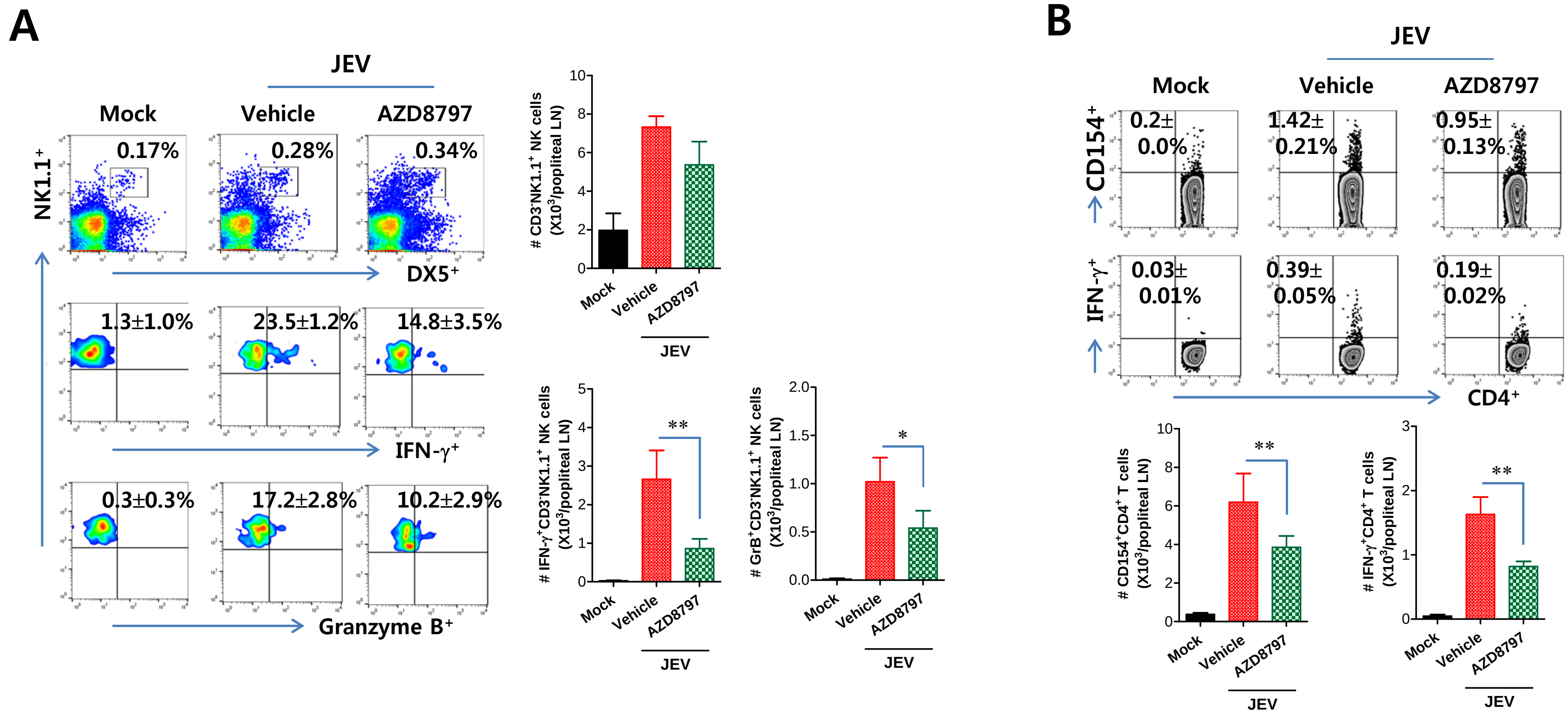

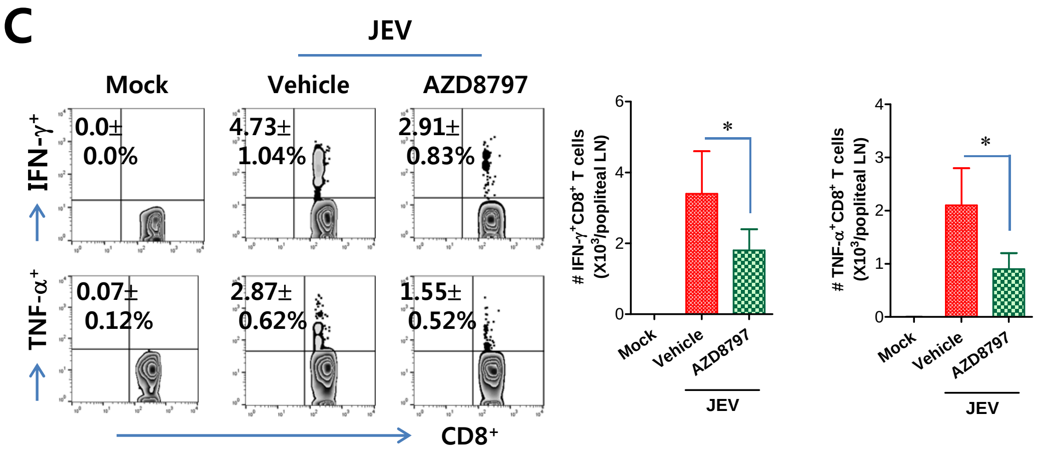

Supplementary Figure 1. Enhanced responses of NK and JEV-specific T cells by CX_3CR1 inhibition. (A) The number and activation of NK cells. After treatment of BL/6 mice with CX_3CR1 inhibitor (AZD8797), the activation of NK cells was determined by enumerating IFN-γ or granzyme B-producing NK cells in popliteal LNs upon brief stimulation with PMA plus ionomycin. Values in dot-plots represent the average ± SEM of IFN-γ or granzyme B-producing cells in $CD3^-NK1.1^+DX5^+$ NK cells. **(B, C)** JEV-specific $CD4^+$ and $CD8^+$ T-cell responses after inhibition of CX_3CR1. Leukocytes were obtained from popliteal LNs from AZD8797-treated BL/6 mice 5 dpi and used for stimulation with JEV epitope peptides of $CD4^+$ T cells ($NS3_{563-574}$) or $CD8^+$ T cells ($NS4B_{215-223}$) for 12 or 8 h, respectively. The frequency and absolute number of JEV-specific $CD4^+$ and $CD8^+$ T cells were determined by intracellular CD154 and IFN-γ staining combined with surface CD4 and CD8 staining. Values in representative dot-plots denote the average ± SEM percentage of indicated cell population. Bar charts show the average ± SEM of values derived from at least two independent experiments (n=3-4). $*p<0.05$; $**p<0.01$ compared between the indicated groups.

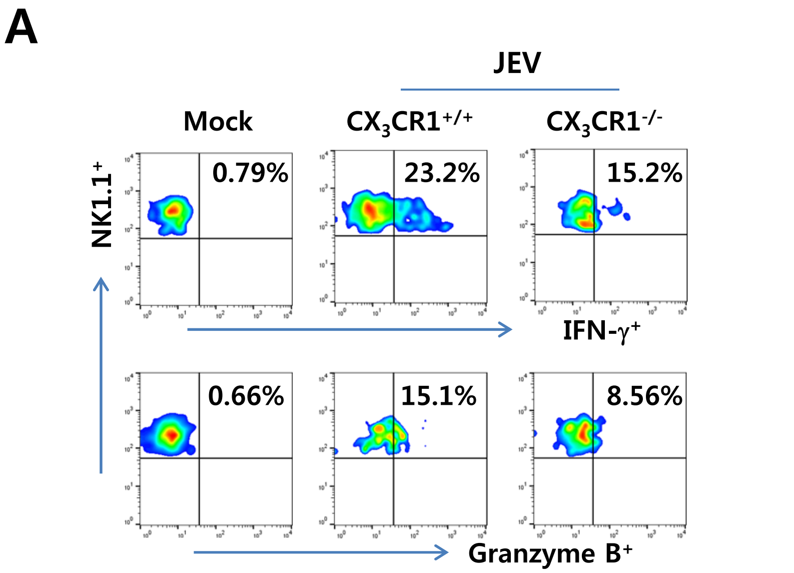

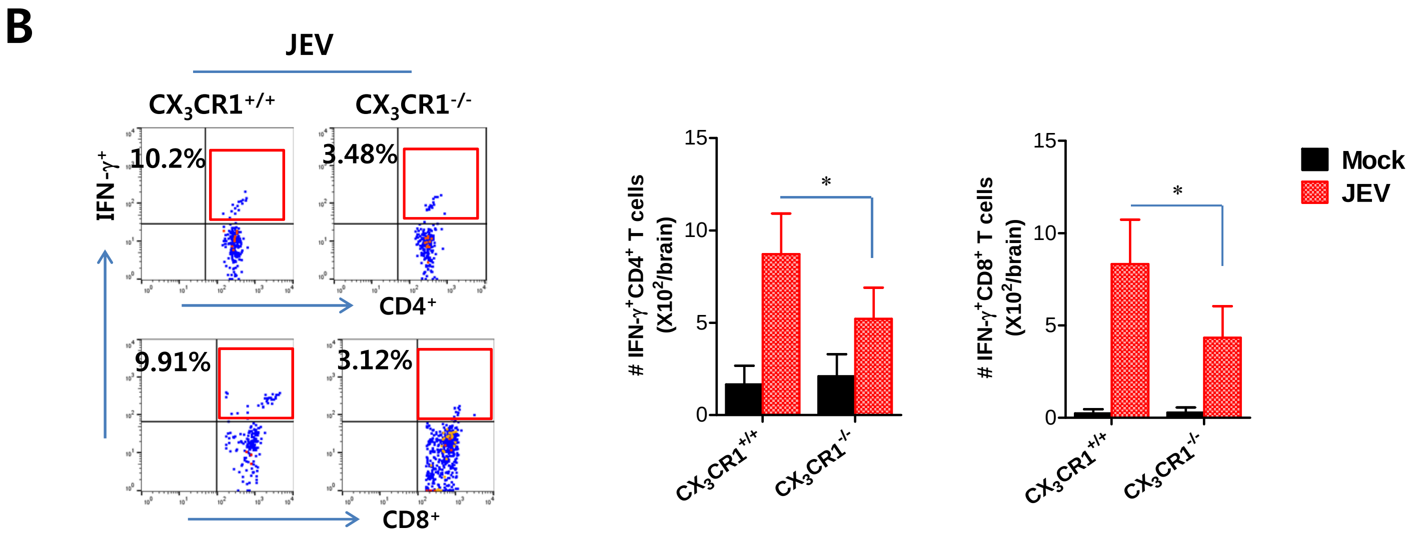

Supplementary Figure 2. The responses of NK and JEV-specific CD4⁺ and CD8⁺ T cells in blood and brain. (A) NK-cell responses in blood. Peripheral blood lymphocytes (PBL) were obtained from $CX_3CR1^{+/+}$ and $CX_3CR1^{-/-}$ mice 2 dpi and briefly stimulated with PMA and ionomcyin. NK cell responses were determined by intracellular IFN-γ and granzyme B staining. Values in dot-plots represent the average percentage of IFN-γ or granzyme B-producing cells in $CD3^-NK1.1^+DX5^+$ NK cells. $CX_3CR1^{+/+}$ mice uninfected with JEV were used for mock-infected group. **(B)** Accumulation of JEV-specific $CD4^+$ and $CD8^+$ T cells in brain. Infiltrated leukocytes were prepared from the brain of surviving $CX_3CR1^{+/+}$ and $CX_3CR1^{-/-}$ mice via vigorous cardiac perfusion and collagenase digestion 7 dpi. JEV-specific $CD4^+$ and $CD8^+$ T cells were detected by intracellular IFN-γ staining in response to stimulation with JEV epitope peptide of $CD4^+$ T cells ($NS3_{563-574}$) or $CD8^+$ T cells ($NS4B_{215-223}$) for 12 or 8 h, respectively. Values in representative dot-plots denote the average percentage of IFN-γ-producing cells in $CD4^+$ or $CD8^+$ T cells. Bar charts show the average ± SEM of values derived from at least two experiments (n=3-4). $*p<0.05$ compared between the indicated groups.

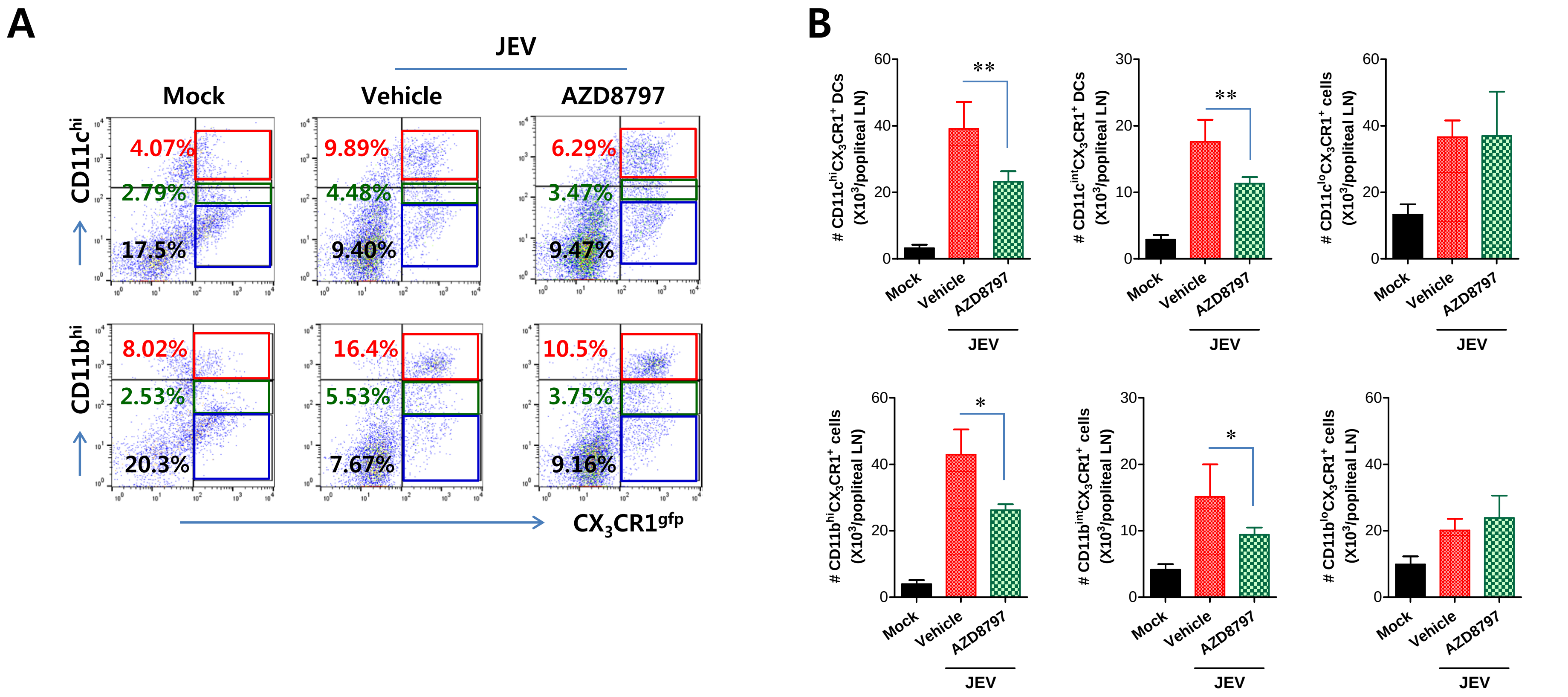

Supplementary Figure 3. CX_3CR1 inhibition regulates the migration of CX_3CR1^+ DCs and $CD11b^+$ myeloid cells in peripheral lymphoid tissues. $CX_3CR1^{+/+}$ wild-type mice were intravenously treated with CX_3CR1 inhibitor (AZD8797, 70 μmol/kg) and infected with JEV via footpad inoculation. CX_3CR1 inhibitor were daily injected to $CX_3CR1^{+/+}$ mice from -1 to 3 dpi. Leukocytes were obtained from popliteal LNs via collagenase digestion at 3 dpi and used to determine CX_3CR1^+ DCs subsets ($CX_3CR1^+CD11c^{hi}$, $CX_3CR1^+CD11c^{int}$), and $CX_3CR1^+CD11c^{lo}$ as well as $CX_3CR1^+CD11b^+$ myeloid cells. **(A)** Values in representative dot-plots denote the average percentage of the indicated cell population after gating on $CD45^+$ cells. **(B)** Bar charts show the average ± SEM of values derived from at least two independent experiments (n=3-4). $*p<0.05$; $**p<0.01$ compared between the indicated groups.
